# Association study of crude seed protein and fat concentration in a USDA pea diversity panel

**DOI:** 10.1002/tpg2.20485

**Published:** 2024-07-31

**Authors:** Renan Uhdre, Clarice J. Coyne, Britton Bourland, Julia Piaskowski, Ping Zheng, Girish M. Ganjyal, Zhiwu Zhang, Rebecca J. McGee, Dorrie Main, Nonoy Bandillo, Mario Morales, Yu Ma, Chengci Chen, William Franck, Adam Thrash, Marilyn L. Warburton

**Affiliations:** ^1^ Department of Crop and Soil Sciences Washington State University Pullman Washington USA; ^2^ USDA ARS Plant Germplasm Introduction and Testing Research Pullman Washington USA; ^3^ Department of Horticulture Washington State University Pullman Washington USA; ^4^ Statistical Programs University of Idaho Moscow Idaho USA; ^5^ School of Food Science Washington State University Pullman Washington USA; ^6^ USDA ARS Grain Legume Genetics and Physiology Research Pullman Washington USA; ^7^ Department of Plant Sciences North Dakota State University Fargo North Dakota USA; ^8^ Department of Horticulture and Crop Science The Ohio State University Columbus Ohio USA; ^9^ Eastern Agriculture Research Center Montana State University Sidney Montana USA; ^10^ Institute for Genomics, Biocomputing & Biotechnology Mississippi State University Mississippi State Mississippi USA

## Abstract

Pea (*Pisum sativum* L.) is a key rotational crop and is increasingly important in the food processing sector for its protein. This study focused on identifying diverse high seed protein concentration (SPC) lines in pea plant genetic resources. Objectives included identifying high‐protein pea lines, exploring genetic architecture across environments, pinpointing genes and metabolic pathways associated with high protein, and documenting information for single nucleotide polymorphism (SNP)‐based marker‐assisted selection. From 2019 to 2021, a 487‐accession pea diversity panel, More protein, More pea, More profit, was evaluated in a randomized complete block design. DNA was extracted for genomic analysis via genotype‐by‐sequencing. Phenotypic analysis included protein and fat measurements in seeds and flower color. Genome‐wide association study (GWAS) used multiple models, and the Pathways Association Study Tool was used for metabolic pathway analysis. Significant associations were found between SNPs and pea seed protein and fat concentration. Gene *Psat7g216440* on chromosome 7, which targets proteins to cellular destinations, including seed storage proteins, was identified as associated with SPC. Genes *Psat4g009200*, *Psat1g199800*, *Psat1g199960*, and *Psat1g033960*, all involved in lipid metabolism, were associated with fat concentration. GWAS also identified genes annotated for storage proteins associated with fat concentration, indicating a complex relationship between fat and protein. Metabolic pathway analysis identified 20 pathways related to fat and seven to protein concentration, involving fatty acids, amino acid and protein metabolism, and the tricarboxylic acid cycle. These findings will assist in breeding of high‐protein, diverse pea cultivars, and SNPs that can be converted to breeder‐friendly molecular marker assays are identified for genes associated with high protein.

## Core Ideas

- Forty‐one diverse accessions were identified with high seed protein concentration.
- Genes, pathways, and markers associated with increased seed protein and fat concentration were identified.
- Genes annotated for storage proteins were associated with fat concentration, indicating interrelated traits.
- Combining genome‐wide association study (GWAS) and Pathways Association Study Tool is a powerful approach to identify candidate genes in pea genomics.
- Using flower color as fixed effect in GWAS removed potential false positive associations with protein concentration.

**Abbreviations**
BLINK: Bayesian‐information and Linkage‐disequilibrium Iteratively Nested Keyway
BLUP: best linear unbiased prediction
CF: Central Ferry
FarmCPU: fixed and random model circulating probability unification
FDR: false discovery rate
GBS: genotype‐by‐sequencing
GWAS: genome‐wide association study
KASP: kompetitive allele specific PCR
LD: linkage disequilibrium
MLM: mixed linear model
MTAs: marker trait associations
NIR: near‐infrared spectrometry
PAST: Pathways Association Study Tool
PGR: pea plant genetic resources
*QQ*
: quantile–quantile
QTL: quantitative trait loci
SNPs: single nucleotide polymorphisms
SPC: seed protein concentration
TCA: tricarboxylic acid cycle

## 1 INTRODUCTION

Pea (*Pisum sativum* L) is an important rotation crop in North America and in other production regions of the world (FAO, 2021b). Pea is beneficial in cereal rotations (Babulicová, 2016), and the average acreage planted in the United States has increased 26% over the past 10 years. Production has expanded to include more states and regions (USDA‐NASS, 2022), partly due to expanded pea protein markets in the food processing industry (Daba & Morris, 2022). Breeding for higher pea seed protein concentration (SPC) is a priority for public and private breeding programs (Daba et al., 2022). Pea plant genetic resources (PGR) are an important source of genetic diversity in modern pea breeding programs (Smýkal et al., 2015) and in genomics assisted breeding (Mahajan et al., 2023; Sharma et al., 2020). Pea protein in new food products ignited a surge in pea PGR distributions from the USDA ARS Plant Germplasm Introduction and Testing Research Unit collections, with 2x or 3x per annum increases resulting in +43,000 seed packets distributed over a 6‐year period (2018–2023; https://www.ars‐grin.gov/).

Pea SPC ranges from 15% to 30% and includes 11S legumin and 7S vicilin globulins (up to 80% of the total seed storage proteins) and albumins (ranging from 26% to 52%) (Robinson et al., 2019; Wang et al., 2003). Globulins are nutritionally deficient in the essential amino acids cysteine and methionine but have physicochemical properties that are important for use in processed foods (Wang et al., 2003). The range in pea seed protein composition enables various food processing options for the plant‐based protein market (Boukid, 2021; Tzitzikas et al., 2006). Increasing the genetic diversity in breeding lines for protein concentration or specific protein composition may allow further gains to be made in the future. Other seed components influence protein levels as well. The accumulation of seed storage proteins in soybeans depends upon triacyl glycerides (which are formed by linking fatty acids to alcohol groups in glycerol). Thus, the same pool of inputs and the same regulatory genes lead to the accumulation of either oils or proteins in legume seeds. This may lead to a negative correlation for levels of these nutritional compounds in the seeds of leguminous crops (Kim et al., 2023).

Large surveys of USDA pea PGR have illuminated the range of pea SPC available in over 2380 gene bank accessions (Search Accessions GRIN‐Global [ars‐grin.gov]; Áli‐Khan & Youngs, 1973; Coyne et al., 2005; Jermyn & Slinkard, 1977). The crude protein in these studies was estimated from the total N using either the Kjeldahl method or Dumas method of analysis (Áli‐Khan & Youngs, 1973; Coyne et al., 2005), respectively. The higher throughput near‐infrared spectrometry (NIR) method has been widely adapted for pea SPC studies (e.g., Burstin et al., 2007). NIR calibration uses total seed N as the proxy for seed protein. While 5.44 would be a more accurate N to protein conversion factor based on amino acid composition in pea seed (FAO, 2021a; Mossé & Huet, 1990), the vast majority of pea SPC publications have used a conversion factor of 6.25.

Genetic studies of pea SPC can be traced back to 1968 (Hynes, 1968; summarized in a review by Casey [1982]). Studies of quantitative trait loci (QTL), pQTL, meta‐QTL, and one genome‐wide association study (GWAS) have been more recently published on pea SPC (Bourgeois et al., 2011; Burstin et al., 2007; Gali et al., 2019; Klein et al., 2014; Tar'an et al., 2004). A summary of these QTL results (Robinson & Domoney, 2021) noted that pea SPC is a complex, quantitative trait with a strong environmental influence, similar to other legumes (Bourgeois et al., 2009; Santalla et al., 2001; Tao et al., 2017). Recently, genomics tools such as genotyping‐by‐sequencing (GBS), a high‐density (90K) single nucleotide polymorphism (SNP) array, and exome capture sequencing have become available and have been used for genotyping pea PGR collections (Aubert et al., 2023; Bari et al., 2021; Zhou et al., 2022). These tools are used to unravel the genetic architecture of complex traits in peas and will aid in future breeding efforts.

Metabolic pathway analysis of an association study can be used to identify the complex network responsible for quantitative traits (Tang et al., 2015). Pathway analysis involves transferring the effects and probabilities attributed to each SNP from a GWAS to linked genes and then assigning these genes to metabolic pathways using information from genomic databases. By assigning the effects and probabilities of association for each SNP to the genes within a pathway and calculating an overall enrichment score for the pathway, researchers can assess the pathway's association with the specific trait of interest. Notably, a recent advancement in this area is the introduction of the Pathways Association Study Tool (PAST), which enables the rapid identification of genes and pathways associated with traits (Thrash et al., 2020), rather than simply listing highly associated SNPs, which is the typical output of a GWAS. While PAST has been extensively used in maize and other outcrossing crop species, it has not yet been used in pea.

Based on these premises and seeking to fill a gap in the literature, this study aims to discover new sources of high pea SPC in a large yellow pea PGR diversity panel, to explore the genetic architecture of pea SPC measured in different environments, to use pathway analysis to identify genes and pathways associated with high protein in pea, and to identify SNPs that can be developed into user‐friendly kompetitive allele specific PCR (KASP) assays that will be useful for future marker‐assisted selection.

**Core Ideas**

- Forty‐one diverse accessions were identified with high seed protein concentration.
- Genes, pathways, and markers associated with increased seed protein and fat concentration were identified.
- Genes annotated for storage proteins were associated with fat concentration, indicating interrelated traits.
- Combining genome‐wide association study (GWAS) and Pathways Association Study Tool is a powerful approach to identify candidate genes in pea genomics.
- Using flower color as fixed effect in GWAS removed potential false positive associations with protein concentration.

## 2 MATERIALS AND METHODS

### 2.1 Plant material

The 487‐line More protein, More pea, More profit (MP3) diversity panel was created by selecting lines from the USDA pea plant genetic resource collection that had yellow cotyledons (*I* gene) and round seeds (*R* gene) (Table S1; Descriptor detail GRIN‐Global [ars‐grin.gov]). Of the 487 accessions, 212 landrace (*II, RR*) accessions were selected from the Pea Single Plant collection (Cheng et al., 2015) using protein estimations from Coyne et al. (2005). An additional 226 landrace (*II, RR*) accessions were selected from the highest SPCs reported in a 2‐year pea field study with two replications of 876 landrace accessions (Jermyn & Slinkard, 1977). A final 24 cultivars and 25 advanced breeding lines with yellow cotyledons and semi‐leafless phenotypes (caused by the *af* or *afila* gene) were added from the USDA ARS breeding program in Pullman, WA. Flower color for each entry was scored as “0” for white and “1” for any pigmented color.

### 2.2 Field study

The MP3 diversity panel was grown in a randomized complete block design with three replications in the Palouse region of Washington State for 3 years. Thirty seeds per plot were sown in April 2019, 2020, and 2021 on the USDA Central Ferry (CF) farm, Central Ferry, WA (46°39′5.1′′ N, 117°45′45.4′′ W; 198 masl). The CF farm has a Chard silt loam soil (coarse‐loamy, mixed, superactive, mesic Calcic Haploxerolls). Soil samples were taken each year and regionally recommended fertilizer mix for peas was applied prior to planting (Table S2). Preemergence herbicide (a,a,a‐trifluoro‐2,6‐dinitro‐*N*,*N*‐dipropyl‐*p*‐toluidine; Treflan, Dow Chemical) was applied each year. Seeds were pre‐treated with fungicides (mefenoxam [13.3 mL a.i. 45 kg^−1^], fludioxonil [2.4 mL a.i. 45 kg^−1^], and thiabendazole [82.9 mL a.i. 45 kg^−1^]); insecticide (thiamethoxam [14.3 mL a.i. 45 kg^−1^]); and sodium molybdate (16 g 45 kg^−1^) and mechanically drilled into 152‐cm‐long double row plots with 30‐cm center spacing, 152 cm plot to plot distance, and 100 cm between paired plots. Supplemental irrigation was applied through subsurface (15 cm) drip for 10 min day^−1^. Plots were manually harvested at physiological maturity and threshed. Seeds were blown clean and stored at 20°C in an air‐conditioned laboratory prior to protein and fat analyses.

### 2.3 Protein and fat determination

Harvested pea SPC was determined using a near infrared spectrometer (Matrix I, Bruker Co.). Calibration curves for protein and fat were developed using 87 accessions selected from the USDA pea core (Table S3). Following Li and Ganjyal (2017), ground seeds of the 87 accessions were measured using the Dumas combustion method with a protein analyzer (FP‐528, LECO Corporation) following AACCI Approved Method 46‐30.01. Three independent samples were run for each entry. Nitrogen was freed by combustion at 950°C in pure oxygen. The nitrogen was then measured by thermal conductivity detection for 200 g from every plot grown in 2019, 2020, and 2021 by NIR (3x per sub‐sample) to determine protein and fat seed concentrations.

### 2.4 Genotyping‐by‐sequencing

The 487 lines of the diversity panel were grown in the greenhouse at 20°C under 16‐h light, and young leaf tissue was collected from a single plant. DNA was extracted with the DNA extraction kit DNeasy Plant 96 (Qiagen Corp.) and submitted to the genome center at the University of Minnesota. A total of 482 samples passed quality control for one enzyme (*Ape*KI) library construction and GBS (Elshire et al., 2011). FreeBayes software (Garrison & Marth, 2012) was used to call the variants using the pea reference genome Cameor (Kreplak et al., 2019). Processing details are presented in Bari et al. (2021).

### 2.5 Phenotypic data analysis

All analyses were performed in R v4.3 (R Core Team, 2023). Best linear unbiased predictions (BLUPs) of the phenotypic values were calculated for each accession in each individual environment (2019, 2020, and 2021) using a mixed linear model (MLM) using the function *lme*r from the lme4 package (Bates et al., 2014), where the accession, environment, accession‐by‐environment, and block were estimated as random effects, flower colors were estimated as a fixed effect, and the error terms were assumed to be independently and identically distributed.

Spatial dependence was evaluated for protein and fat by fitting empirical variograms for each trial to check for spatial effects using package "gstat" (Gräler et al., 2016; Pebesma, 2004). All semivariograms had a partial sill close to zero and a flat semivariance pattern as distance between plots increased, indicating no detectable spatial covariance. The multiyear analysis was implemented using the following model:

$$Y_{ijkl}=\mu +G_{i}+B\_j+Fc\_k+Yr_{l}+{\left(GY\right)}_{il}+\varepsilon_{ijkl},$$

where $Y_{ijkl}$ is the phenotypic observation, μ is model intercept, $G_{i}$ is the random effect of the *i*th accession, B_j is the random effect of the *j*th block, Fc_k is the fixed effect of the *k*th flower color, $Yr_{l}$ is the random effect of the *l*th environment, ${\left(GY\right)}_{il}$ is the random effect of the *i*th accession in the *l*th environment, and $\varepsilon_{ijkl}\;$ are the error terms. Assumptions regarding normality, independence, and homoscedasticity of error terms were assessed using quantile–quantile (*QQ*)‐plots and plots of residual versus fitted values.

### 2.6 Genome‐wide association study

From the SNPs extracted from the sequencing analysis, a set of 114,687 SNPs was obtained after filtering using the VCFtools software version 0.1.16 (Danecek et al., 2011) with the following criteria: biallelic SNPs; SNPs with mean depth ≥3; SNPs with a missing rate <0.5; SNPs with minor allele frequency >0.05; and marker heterozygosity <0.65. The filtered SNP set was used to perform GWAS and associations were tested using three different models: MLM (Yu et al., 2006), fixed and random model circulating probability unification (FarmCPU) (Liu et al., 2016), and Bayesian‐information and Linkage‐disequilibrium Iteratively Nested Keyway (BLINK) (Huang et al., 2019) with the GAPIT version 3 software package (Wang & Zhang, 2021). A principal component analysis was used to correct for population structure in the models using the first six principal components as covariates. *QQ*‐plots were used to check if the observed *p*‐values from each model deviated from expected *p*‐values, which should have a uniform distribution between 0 and 1. *p*‐values obtained from the associated markers were filtered based on false discovery rate (FDR) <0.05 implemented in GAPIT 3.

### 2.7 Population structure analysis

Population structure analysis was conducted to correct for genetic substructure present in the diversity panel during GWAS. The model‐based clustering algorithm ADMIXTURE (Alexander et al., 2009), coupled with centroid analysis, was used to ascertain the most likely number of subpopulations using all the SNPs from our data set and was performed in multiple runs by inputting successive values of *K* from 3 to 15. A 10‐fold cross‐validation procedure was performed for each *K*‐value to find the most likely one. The *K*‐means clustering, and discriminant analysis further validated the optimal number of K in this diversity panel. For each accession, ADMIXTURE then provides estimates of the probability of membership to each cluster, which creates the *Q* matrix.

### 2.8 Pathway analysis and candidate genes

GWAS outputs from the BLINK model were analyzed using the Pathway Association Study Tool (PAST 2.0.0‐rc) (https://github.com/IGBB/PAST), following the protocols of Thrash et al. (2020). The GWAS data were input into PAST with pathway and gene annotation data from PulseDB (https://www.pulsedb.org/jbrowses). The data encompassed SNP‐trait association values, correlation metrics (*R* ^2^), effect values, and linkage disequilibrium (LD) measurements for each SNP and its neighboring SNPs (50 upstream and 50 downstream), as discussed in Warburton et al. (2022). The association and effect files were used without filtering for statistical significance. For gene assignment, SNPs were associated with genes based on LD and genomic distance between SNPs and genes, following the methodology of Thrash et al. (2020).

The analysis identifies pathways that influence both increases and decreases in trait expression. Only pathways with a minimum of four annotated genes were considered to minimize small sample size biases. The association significance between pathways and phenotypes was determined through the creation of 1000 random gene effect distributions, which were compared against the observed effects. PAST identified genes and pathways for each marker trait association (MTA), unless a linked gene was not annotated with a pathway or was associated with a pathway containing less than four genes. For cases where SNPs were not next to annotated genes in PulseDB, we found all gene models within a 70kb window and referred to their homologs in *Medicago truncatula* or *Arabidopsis thaliana* model species’ genomics databases (https://ensembl.gramene.org/index.html), which provided insights into their potential roles and mechanisms.

## 3 RESULTS

### 3.1 Phenotypic traits and correlations

Seed protein and fat were measured in triplicate for each sample, representing each replication within each year for every entry in the GWAS population. The BLUPs for both traits averaged over replications are presented in Table S4, which also presents flower color. The lines had a high genetic variability for protein and fat, and high heritability estimates (Table 1). The combined analysis of variance indicates that the genotype effects are significantly different between genotype (*p*‐values < 0.05), and the genotype by environment interaction for both traits was highly significant (Table 1).

**TABLE 1:** Variance components for the mixed model analyses of seed protein and fat concentrations in peas grown over 3 years.

Variable	Year							
	2019		2020	2021			Multiyear	
	Protein	Fat	Protein	Fat	Protein	Fat	Protein	Fat
Accession (G)	0.83	0.05	1.35	0.03	1.12	0.06	1.09	0.04
Block	0.14	0.0005	0.11	0.0001	0.11	0.001	0.12^a^	0.03^a^
Environment (E)							0.23	0.07
G × E							0.07	0.09
Residual	2.81	0.02	2.54	0.05	0.68	0.02	2.00	0.03
Heritability							0.79	0.83

^a^ Block within environment.

Pearson correlations were calculated between protein, fat, and flower color traits, which revealed that fat concentration is highly negatively correlated to flower color (*r* = −0.89, *p* ≤ 0.01). Other correlations between traits were low (Table S5). It is readily apparent from the original BLUP data in Table S4 that entries with purple flowers have higher protein and lower fat concentrations than lines with white flowers, which may explain some of this correlation. To correct for this unexpected correlation, flower color was used as a control variable and BLUPs were recalculated (Table S6). This caused a decrease in the confounding effect and corrected correlation information is presented in Table 2. The correlations between fat and protein concentration and fat and flower color are low in the new analysis.

**TABLE 2:** Analysis of Pearson correlations for the traits measured in the USDA pea diversity panel, including protein and fat grown in 3 years (2019, 2020, and 2021), the average over 3 years (MY), and flower color.

	Protein MY	Protein 2019	Protein 2020	Protein 2021	Fat MY	Fat 2019	Fat 2020	Fat 2021	Flower Color
Protein MY	1								
Protein 2019	0.998^*^	1							
Protein 2020	0.997^*^	0.991^*^	1						
Protein 2021	0.998^*^	0.995^*^	0.993^*^	1					
Fat MY	0.111^*^	0.105^*^	0.121^*^	0.107^*^	1				
Fat 2019	0.111^*^	0.111^*^	0.115^*^	0.104^*^	0.975^*^	1			
Fat 2020	0.078	0.072	0.091	0.072	0.947^*^	0.902^*^	1		
Fat 2021	0.129^*^	0.118^*^	0.139^*^	0.130^*^	0.954^*^	0.907^*^	0.829^*^	1	
Flower color	0.004	0.002	−0.001	0.011	−0.102^*^	−0.080	−0.006	−0.197^*^	1

* *p ≤* 0.01.

### 3.2 Structure analysis

The population structure analysis was conducted using the filtered set of 114,687 SNP markers distributed along seven chromosomes (Figure S1) and identified six genetically distinct subpopulations within the 487 diverse pea accessions (Table S7, Figures S2 and S3). All but three of the cultivars clustered together in the first group. The second group was very mixed, with landraces from all over the world, but predominantly from the United States, Europe, and Ethiopia. Landraces from South‐Central Asia clustered into the third group, and the fourth group was mostly composed of landraces from Ethiopia and India, along with a few from other countries. The fifth group was composed mostly of landraces from Europe, Turkey, several former Soviet countries, and a few others, and also two cultivars from the United States. The sixth group was again highly diverse, with landraces from all over the world. For *Pisum sativum* L., this admixture analysis reveals genetic exchange among pea varieties, influenced by factors such as seed exchange between breeding programs and marketing of seed between growing regions. The ancestry proportion estimates in Table S7 for different genetic clusters illuminate population admixture history and current genetic backgrounds. This aligns with prior publications (Bogdanova et al., 2018; Shatskaya et al., 2023).

### 3.3 Genome‐wide association

**FIGURE 1:**
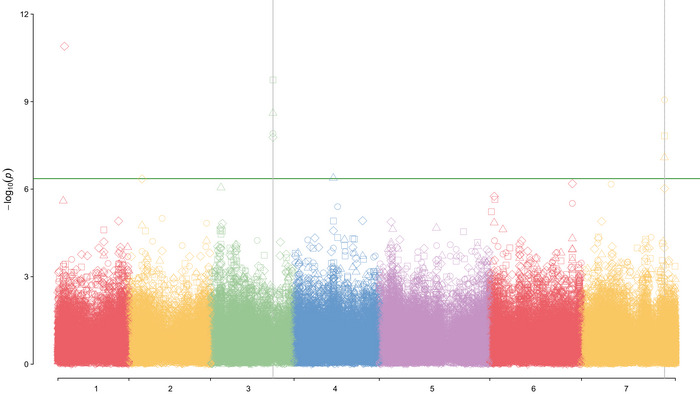
Manhattan plot of *p*‐values for marker‐trait associations analysis with the Bayesian‐information and Linkage‐disequilibrium Iteratively Nested Keyway (BLINK) model for seed protein concentration in 2019, 2020, 2021, and over years (multiyears) evaluations. The vertical axis shows the significance of association with the threshold chosen for this study marked by the continuous green line. The horizontal axis shows the chromosomal location of each single‐nucleotide polymorphism for the seven pea chromosomes. ○: Protein 2019; ∆: Protein 2020; ◊: Protein 2021; □: Protein multiyear.

**FIGURE 2:**
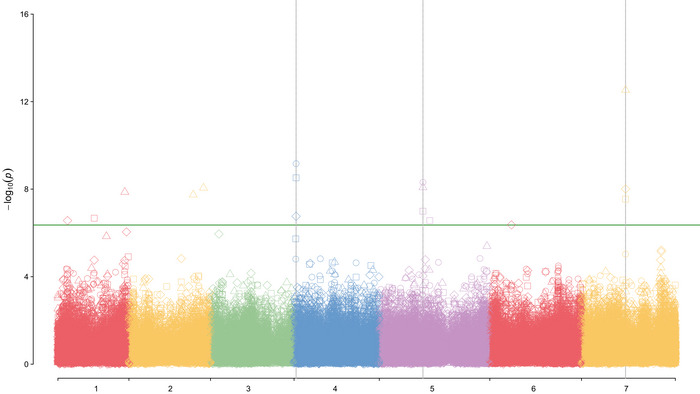
Manhattan plot of *p*‐values for marker‐trait associations analysis with the Bayesian‐information and Linkage‐disequilibrium Iteratively Nested Keyway (BLINK) model for seed fat concentration 2019, 2020, 2021, and over years (multiyears). The vertical axis shows the significance of association with the threshold chosen for this study marked by the continuous green line. The horizontal axis shows the chromosomal location of each single‐nucleotide polymorphism for the seven pea chromosomes. ○: Fat 2019; ∆: Fat 2020; ◊: Fat 2021; □: Fat multiyears.

MTA analysis was performed using all models (MLM, FarmCPU, and BLINK) by year with all traits. The *p*‐values obtained from MTAs were corrected based on FDR < 0.05. *QQ*‐plots are presented in Figure S4. All models analyzing all traits appeared to have a good fit for the data and sharp deviation from the expected *p*‐value, indicating adequate control of false positives (Mugabe et al., 2023). However, the fit for the BLINK model appears to be the best, and these data were used in subsequent analyses and are presented in Table 3. Association data for the MLM and FarmCPU models are shown in Table S8. Genome‐wide association analysis of SPC with BLINK identified four significantly (*p* < 4.36E‐07) associated SNPs on chromosomes 1, 3, 4, and 7 (Figure 1, Table 3), explaining 1%–13.34% of the phenotypic variation. Eleven different MTAs were identified for seed fat concentration on chromosomes 1, 2, 4, 5, 6, and 7, explaining 1.2%–26.30% of the phenotypic variation. The significant MTAs for all traits and models can be found in Figure S5 and Table S8. SNP S3LG5_327115929 on chromosome 3 was associated with SPC in all years and the multiyear analysis (Figure 1, Table 3), and SNP S4LG4_10586992 on chromosome 4 was associated with seed fat concentration in 2019 and 2021 and the multiyear analysis (Figure 2, Table 3). Figures S6 and S7 represent the total phenotypic variation in protein and fat concentration explained by the six groups identified, but there was no distinct pattern, and no single group was significantly different for either trait.

**TABLE 3:** List of significant single nucleotide polymorphisms (SNPs) associated with seed protein and fat concentration in pea identified using the Bayesian‐information and Linkage‐disequilibrium Iteratively Nested Keyway (BLINK) model in the USDA pea diversity panel.

Traits	Years	SNP ID	Chr	Position	Allele	*p*‐value	Effect^a^	MAF	PVE (%)
Protein	MY	S3LG5_327115929	3	327115929	C/T	1.80E‐10	0.299	0.136	11.86
	MY	S7LG7_434876919	7	434876919	A/G	1.50E‐08	−0.183	0.238	4.32
	2019	S3LG5_327115929	3	327115929	C/T	1.24E‐08	0.247	0.136	11.25
	2019	S7LG7_434876919	7	434876919	A/G	8.64E‐10	−0.206	0.238	4.28
	2020	S3LG5_327115929	3	327115929	C/T	2.48E‐09	0.261	0.138	8.65
	2020	S4LG4_206013520	4	206013520	A/G	4.18E‐07	−0.165	0.215	1.01
	2020	S7LG7_434876919	7	434876919	A/G	8.24E‐08	−0.186	0.239	2.90
	2021	S1LG6_34341647	1	34341647	C/A	1.27E‐11	−0.325	0.111	5.82
	2021	S3LG5_327115929	3	327115929	C/T	1.68E‐08	0.248	0.136	13.34
Fat	MY	S1LG6_190292106	1	190292106	G/A	2.13E‐07	0.031	0.338	1.54
	MY	S4LG4_10586992	4	10586992	C/G	3.02E‐09	0.058	0.277	2.14
	MY	S5LG3_228836884	5	228836884	G/A	1.02E‐07	−0.063	0.057	8.69
	MY	S5LG3_264821413	5	264821413	C/A	2.73E‐07	−0.057	0.138	3.27
	MY	S7LG7_230618540	7	230618540	G/A	2.86E‐08	−0.060	0.084	5.53
	2019	S4LG4_10586992	4	10586992	C/G	6.82E‐10	0.064	0.277	3.49
	2019	S5LG3_228836858	5	228836858	C/G	4.87E‐09	0.073	0.057	26.30
	2020	S1LG6_350543492	1	350543492	A/G	1.36E‐08	0.131	0.081	13.32
	2020	S2LG1_336760270	2	336760270	A/C	1.80E‐08	−0.047	0.239	1.73
	2020	S2LG1_390717452	2	390717452	G/C	8.71E‐09	0.040	0.393	1.28
	2020	S5LG3_228836884	5	228836884	G/A	8.38E‐09	−0.071	0.057	5.46
	2020	S7LG7_230618540	7	230618540	G/A	2.96E‐13	−0.096	0.079	7.78
	2021	S1LG6_50302727	1	50302727	G/A	2.73E‐07	0.087	0.061	4.00
	2021	S4LG4_10586992	4	10586992	C/G	1.74E‐07	0.055	0.278	1.23
	2021	S6LG2_113204819	6	113204819	C/T	4.29E‐07	−0.051	0.291	3.55
	2021	S7LG7_230618540	7	230618540	G/A	9.76E‐09	−0.068	0.083	9.10

*Note*: Protein and fat were measured from seeds grown in 2019, 2020, and 2021, and averaged over years (MY).

Abbreviations: Chr, chromosome; MAF, minor allele frequency; PVE (%), percentage of phenotypic variance explained.

^a^ Effect associated with the allele in reverse alphabetic order.

The gene associations found using GWAS are within the LD breakdown distances from the SNPs causing the MTAs. A study by Siol et al. (2017) found LD decays to an *R* ^2^ value less than 0.8 within ∼200 kb in a population of pea cultivars and 100 kb in wild or landrace pea genotypes. The breakdown of LD is not constant across the genome; however, data from the present study show that the median breakdown of LD distance below an *R* ^2^ value of 0.8 ranges from 2362 bp on chromosome 2 to 3,000,964 bp on chromosome 4 and an overall median of 51,835 bp across chromosomes. However, pairs of SNPs linked with an *R* ^2^ value less than 0.8 range from 0 bp (adjacent) to 562,176,108 bp apart. While the distribution of linkages skews toward the shorter physical distances, this indicates that there are very large linked blocks in our data set (Table S9 and data not shown). In this study, potentially associated genes were identified within a window of 140 kb (±70 kb) of the associated SNP positions listed in Table 3. This search yielded 56 possible candidate genes (Table 4).

**TABLE 4:** List of candidate genes identified in the study.

SNP ID	Chr	SNP position	Trait	Gene name	Gene distance (bp)	Gene position	Biological function
S1LG6 190292106	1	190292106	Fat MY	*Psat1g104000*	7142	190299248	Glycoside hydrolase + catalytic core
				*Psat1g104040*	89,314	190381420	Unknown gene
S4LG4 10586992	4	10586992	Fat MY	*Psat4g009240*	−8572	10578420	Formin Homology 2 domain
			Fat 2019	*Psat4g009280*	10,743	10597735	NLI interacting factor‐like phosphatase
			Fat 2021	*Psat4g009320*	23,058	10610050	Unknown gene
				*Psat4g009200*	−84,656	10502336	Lipocalin‐like domain
S5LG3 228836884	5	228836884	Fat MY	*Psat5g127280*	−2845	228834039	Sedoheptulose‐1 + 7‐bisphosphatase family signature
			Fat 2020	*Psat5g127240*	−23,868	228813016	Protein of unknown function + DUF573
				*Psat5g127200*	−46,507	228790377	Unknown gene
				*Psat5g127160*	−58,235	228778649	Unknown gene
				*Psat5g127120*	−79,730	228757154	Transferase activity + transferring acyl groups other than amino‐acyl groups
				*Psat5g127320*	38,330	228875214	Unknown gene
				*Psat5g127360*	49,527	228886411	HAD superfamily + subfamily IIIB (acid phosphatase)
				*Psat5g127400*	51,392	228888276	Myb/SANT‐like DNA‐binding domain
				*Psat5g127440*	98,739	228935623	Transport protein particle (TRAPP) component
S5LG3 228836858	5	228836858	Fat 2019	*Psat5g127280*	−2819	228834039	Sedoheptulose‐1 + 7‐bisphosphatase family signature
				*Psat5g127240*	−23,842	228813016	Protein of unknown function + DUF573
				*Psat5g127200*	−46,481	228790377	Unknown gene
				*Psat5g127160*	−58,209	228778649	Unknown gene
				*Psat5g127120*	−79,704	228757154	Transferase activity + transferring acyl groups other than amino‐acyl groups
				*Psat5g127320*	38,356	228875214	Unknown gene
				*Psat5g127400*	51,418	228888276	Myb/SANT‐like DNA‐binding domain
				*Psat5g127360*	49,553	228886411	Haloacid Dehydrogenase (HAD) superfamily + subfamily IIIB (acid phosphatase)
				*Psat5g127440*	98,765	228935623	Transport protein particle (TRAPP) component
S5LG3 264821413	5	264821413	Fat MY	*Psat5g145760*	−4233	264817180	Unknown gene
				*Psat5g145720*	−6659	264814754	Protein of unknown function (DUF1191)
				*Psat5g145680*	−49,056	264772357	Prolamin‐like
S7LG7 230618540	7	230618540	Fat MY	*Psat7g136600*	−7853	230610687	Protein tyrosine kinase
			Fat 2020	*Psat7g136520*	−135,698	230482842	Protein modification by small protein conjugation or removal
			Fat 2021				
S1LG6 350543492	1	350543492	Fat 2020	*Psat1g199800*	−2756	350540736	GDSL‐like lipase/acylhydrolase family
				*Psat1g199880*	37,986	350581478	Aspartic acid proteinase inhibitor
				*Psat1g199960*	82,332	350625824	GDSL/SGNH‐like acyl‐esterase family found in Pmr5 and Cas1p
				*Psat1g199760*	−39,243	350504249	Unknown gene
				*Psat1g199720*	−43,300	350500192	Tho complex subunit 7
				*Psat1g199680*	−73,395	350470097	Pentatricopeptide repeat (PPR)
				*Psat1g199640*	−74,158	350469334	Unknown gene
				*Psat1g199560*	90,509	350452983	Thiamine pyrophosphate enzyme + N‐terminal TPP binding domain
S2LG1 336760270	2	336760270	Fat 2020	*Psat2g129400*	−447	336759823	Unknown gene
				*Psat2g129360*	−51,596	336708674	Glutathione S‐transferase + N‐terminal domain
				*Psat2g129320*	−73,844	336686426	Proline‐rich extensin signature
S2LG1 390717452	2	390717452	Fat 2020	*Psat2g159120*	7379	390724831	GATA zinc finger
				*Psat2g159080*	−23,146	390694306	HhH‐GPD superfamily base excision DNA repair protein
				*Psat2g159040*	−41,121	390676331	Regulation of cellular nucleobase + nucleoside + nucleotide and nucleic acid metabolic process
S1LG6 50302727	1	50302727	Fat 2021	*Psat1g033920*	86,275	50389002	MatE
				*Psat1g033960*	167,300	50470027	Probable lipid transfer
S6LG2 113204819	6	113204819	Fat 2021	*Psat6g081400*	−512	113204307	Eukaryotic‐type carbonic anhydrase
				*Psat6g081360*	−12,884	113191935	Unknown gene
				*Psat6g081320*	−40,772	113164047	Cyclin + N‐terminal domain
S3LG5 327115929	3	327115929	Protein MY	*Psat3g164360*	−589	327115340	SQUAMOSA binding Protein (SBP) domain
			Protein 2019				
			Protein 2020				
			Protein 2021				
S7LG7 434876919	7	434876919	Protein MY	*Psat7g216440*	−589	434876330	Sec23/Sec24 trunk domain
			Protein 2019	*Psat7g216480*	36,426	434913345	Unknown gene
			Protein 2020	*Psat7g216520*	50,027	434926946	U‐box domain
			Protein 2021	*Psat7g216400*	−66,149	434810770	Polyketide cyclase/dehydrase
				*Psat7g216360*	−76,291	434800628	Polyketide cyclase/dehydrase
				*Psat7g216280*	−94,635	434782284	Response regulator receiver domain
S4LG4 206013520	4	206013520	Protein 2020	*Psat4g110320*	−9948	206003572	Unknown gene
				*Psat4g110280*	−23,269	205990251	Unknown gene
				*Psat4g110240*	−41,217	205972303	Regulation of biological process
				*Psat4g110200*	−80,309	205933211	Protein kinase domain
				*Psat4g110400*	16,055	206029575	Transcription factor regulating root and shoot growth via Pin3
				*Psat4g110440*	39,752	206053272	Unknown gene
S1LG6 34341647	1	34341647	Protein 2021	*Psat1g024680*	−14,927	34326720	Nuclear pore complex scaffold + nucleoporins 186/192/205
				*Psat1g024720*	32,679	34374326	Heme oxygenase
				*Psat1g024760*	81,874	34423521	Metalloenzyme superfamily

*Note*: Significant single nucleotide polymorphisms (SNPs) associated with seed protein and fat concentration in pea identified using Bayesian‐information and Linkage‐disequilibrium Iteratively Nested Keyway (BLINK) model in the USDA pea diversity panel are listed with reported candidate genes within ±70 kb of SNPs and reported annotation information.

Abbreviations: Chr, chromosome; MY, averaged over years.

### 3.4 Pathway analysis

For the pathway analysis, the window around the associated SNPs was reduced to only the most tightly linked, using calculated LD data, to identify the most likely associated gene(s) based on available data (Thrash et al., 2020). At a significance threshold of *p* < 0.02, the PAST pathway analysis identified seven pathways associated with SPC and 20 pathways associated with seed fat concentration using the BLINK multiyear analysis (Table 5). A total of 76 pathways were associated at *p* < 0.05, and although this is too large a number to be helpful in determining possible mechanisms for the traits under study, some interesting patterns were seen. For example, the pathways PWY‐5120 (geranylgeranyl diphosphate biosynthesis) and SALVADEHYPOX‐PWY (adenosine nucleotides degradation II) were found in all years and for the average over years (MY) analysis of SPC, and the pathways PWY‐7511 (protein ubiquitination) and PWY‐5723 (Rubisco shunt) were found in all years and for the multiyear analysis of seed fat concentration (Table S10).

**TABLE 5:** Metabolic pathways associated (*p* < 0.02) with protein and fat averaged over years (MY) traits following analysis of genome‐wide association study results using the program Pathways Association Study Tool.

Trait	Pathway ID	Pathway description	*p*‐value	Gene	Model
Protein	LIPAS‐PWY	Triacylglycerol degradation	0.001638	*Psat5g108360*	Dec.
	NAGLIPASYN‐PWY	Lipid IV_A_ biosynthesis	0.005724	*Psat6g046400*	Dec.
	LEU‐DEG2‐PWY	l‐leucine degradation I	0.006610	*Psat7g125400*	Dec.
	PWY‐5120	Geranylgeranyl diphosphate biosynthesis	0.006897	*Psat1g221200*	Dec.
	PWY‐6361	1D‐*myo*‐inositol hexakisphosphate biosynthesis I (from Ins(1,4,5)P3)	0.014051	*Psat6g009400*	Inc.
	PWY‐6317	d‐galactose degradation I (Leloir pathway)	0.014934	*Psat7g020320*	Inc.
	PWY66‐422	d‐galactose degradation V (Leloir pathway)	0.015966	*Psat7g020320*	Inc.
Fat	PWY‐5918	Superpathay of heme b biosynthesis from glutamate	0.000347	*Psat6g156560*	Dec.
	PWY‐7229	Superpathway of adenosine nucleotides de novo biosynthesis I	0.001104	*Psat2g185280*	Dec.
	PWY66‐21	Ethanol degradation II	0.00138	*Psat5g112840*	Dec.
	HEME‐BIOSYNTHESIS‐II	Heme b biosynthesis I (aerobic)	0.002542	*Psat3g180200*	Dec.
	PWY‐841	Superpathway of purine nucleotides de novo biosynthesis I	0.00280	*Psat2g068800*	Dec.
	PWY‐1042	Glycolysis IV (plant cytosol)	0.007014	*Psat5g295240*	Dec.
	PWY‐7214	Baicalein degradation (hydrogen peroxide detoxification)	0.008892	*Psat5g066280*	Dec.
	PWY‐7200	Superpathway of pyrimidine deoxyribonucleoside salvage	0.009243	*Psat2g185280*	Dec.
	PWY‐7219	Adenosine ribonucleotides de novo biosynthesis	0.009369	*Psat4g218800*	Dec.
	PWY‐5723	Rubisco shunt	0.009519	*Psat6g212720*	Dec.
	PWY‐5971	Palmitate biosynthesis II (bacteria and plants)	0.009934	*Psat4g011560*	Dec.
	SALVADEHYPOX‐PWY	Adenosine nucleotides degradation II	0.010418	*Psat2g033440*	Inc.
	PWY‐5989	Stearate biosynthesis II (bacteria and plants)	0.010816	*Psat4g011560*	Inc.
	PWY‐7445	Luteolin triglucuronide degradation	0.011202	*Psat5g066280*	Inc.
	PWY‐2501	Fatty acid & alpha; ‐oxidation I	0.011416	*Psat1g040320*	Inc.
	PWY‐5156	Superpathway of fatty acid biosynthesis II (plant)	0.013146	*Psat4g011560*	Inc.
	PWY‐6803	Phosphatidylcholine acyl editing	0.014330	*Psat4g011560*	Inc.
	PWY‐7511	Protein ubiquitination	0.017878	*Psat5g284440*	Inc.
	PWY‐5350	Thiosulfate disproportionation IV (rhodanese)	0.017907	*Psat1g013760*	Inc.
	PWY‐7033	Alkane biosynthesis II	0.019048	*Psat4g011560*	Inc.

*Note*: Pathways are presented that are associated with an increase (Inc.) or decrease (Dec.) in the expression of the traits. The pathway IDs and descriptions are from the PulseDB or Gramene databases.

## 4 DISCUSSION

The wide range of diversity in seed protein and fat concentration displayed in this study enabled the identification of potential donor lines for future breeding activities to create diverse new high‐protein pea cultivars. Table S4 lists 38 purple‐flowered and three white‐flowered accessions or single‐plant‐derived lines from accessions with over 23% seed protein averaged over the 3 years. Nearly all of these 41 lines had over 22% SPC every year of the study, showing that they are consistently high‐protein lines in different growing environments, representing new genetic diversity that may allow further genetic gain via selection.

To test the power of the current data set to identify genes via GWAS and known genetic mechanisms via the PAST pathway analysis, which has not yet been reported in pea, simply inherited traits were sought that could be scored on the plants in the panel. The only simply inherited Mendelian trait segregating in this data set that was recorded was flower color (purple or pigmented vs. white) (Table S4). Thus, GWAS was run on flower color to test the GBS data and the models run. The main MTA found for the flower color trait in all models was S6LG2_68261112, which was ∼70K bp from *Psat6g060480*, the gene that encodes the BHLH‐MYC transcription factor that is now known to be the gene that Mendel first identified with his pioneering pea flower color trait study (Hellens et al., 2010). One of the significantly associated SNPs in the MLM model is within 663 bp of the gene, which starts at 68,330,158 bp on chromosome 6.

Previously published studies of LD breakdown in pea indicated that LD decays to *R* ^2^ ≤ 0.8 in pea within 100–200 kb on average (Siol et al., 2017). Although we know that even larger LD blocks exist in this panel of individuals, we chose to look for candidate genes within a distance of ±70 kb because the main flower color MTA was ∼67 kb from the known causal gene. Unfortunately, *Psat6g060480* is in one of these large linkage blocks, and the effect of the gene on flower color is so strong that under the MLM model, the significantly associated SNPs span just over 2 million base pairs. The BLINK and FarmCPU models did not identify more than one SNP associated with flower color and thus were able to avoid the wide LD block. The effect of the long LD blocks will make accurate GWAS and successful use of the PAST analysis, prone to false positive results in this data set, and in inbreeding species in general. Running and comparing more than one GWAS model may help reduce the uncertainty around which gene is causing the MTA but will continue to be a problem in inbreeding species with slow LD decay.

The original BLUP analysis found that the flower color MTA affected not only flower color but was also strongly associated with protein and fat concentrations. The correlations originally seen between the three traits (Table S5) could have been caused by subpopulation structure in the analysis, since many of the purple lines were high protein and, to a lesser extent, lower fat lines (Table S4). The correlations could also have been caused by the very large LD block around the flower color gene, which may also contain genes influencing fat and protein, and it may have been a combination of these and the fact that seed composition traits are often correlated (Abdel‐Aal et al., 2019; Santos et al., 2019).

One MTA that may be causing a correlation between fat and flower color due to a large linkage block is seen in the GWAS using BLUPs uncorrected for flower color. A large effect MTA at SNP S6LG2_67037282 was found for seed fat concentration, very close to the flower color gene on chromosome 6. The SNP was most closely linked to *Psat6g059760*, and this gene is described in PulseDB as a probable lipid transfer gene. In the model organism *Arabidopsis thaliana*, a BLAST comparison identified the homologous gene *AT3G22600*, which encodes a bifunctional inhibitor/lipid‐transfer protein/seed storage 2S albumin superfamily protein. This gene is known to be important in the accumulation of lipids and fatty acids in plants, and specifically of seed oil concentration in soybean (Qi et al., 2018) and rice (Wang et al., 2015). It was also found in a GWAS of seed fat concentration in chickpea, where it was not confounded with flower color (Sari et al., 2024).

To remove the effect of flower color on protein and fat, whether caused by linkage or population substructure, the corrected BLUP analysis was run. The MTAs found for corrected protein and fat BLUPs using the BLINK model do not include any in the flower color linkage block on chromosome 6, nor do any of the MTAs identified with the BLUPs run without correcting for flower color. The new MTAs are linked to between one and nine genes, each within a window of ±70 kb. For genomic regions where we found the LD extended further than this, we did look further upstream and downstream to find the genes reported in Table 4. The genes identified for SPC include *Psat7g216440* on chromosome 7, closely linked to SNP S7G7_434876919, which influenced SPC in 2019, 2020, and the multiyear analysis. This gene contains a Sec23/Sec24 trunk domain, which targets newly created proteins to their final cellular location, including seed storage proteins.

The genes linked to MTAs identified for seed fat concentration included *Psat4g009200*, linked to SNP *S4LG4_10586992*, an MTA identified in 2019, 2021, and the multiyear analysis, which encodes a lipocalin, a gene involved in lipid metabolism. In addition, *Psat1g199800* and *Psat1g199960* are both lipases linked to MTA S1LG6_350543492, identified in 2020. Lipases break down lipids and could reduce the amount of lipids found in the seed. Gene *Psat1g033960*, linked to S1LG6_50302727, identified in 2020, is a lipid transfer gene and influences final lipid concentration as well. Interestingly, genes that would be excellent seed protein candidates were identified in the seed fat GWAS, including *Psat5g127440*, linked to MTA S5LG3_228836858, which also targets newly created proteins to their final cellular location; and *Psat5g145680*, linked to MTA S5LG3_264821413, which creates prolamin seed storage proteins. The identification of genes apparently influencing SPC in the fat GWAS may simply be a mistake due to linkage drag of unrelated genes but it may also indicate the interrelationship of seed storage components.

Past research using bi‐parental mapping populations and one recombinant inbred line population have identified QTLs for similar protein and fat levels in pea seeds (Bourgeois et al., 2011; Burstin et al., 2007; Gali et al., 2024, 2019; Klein et al., 2014; Tar'an et al., 2004; Zhou et al., 2022). More MTAs were identified in the current study than in the previous mapping studies, probably due to the broader genetic diversity of the MP3 panel than the few parents of the populations. The identified MTAs of the current study were not within ∼1.6 million base pairs of any QTL identified in these earlier studies. This suggests that the MTAs reported here are novel and may help to further deepen our understanding of the genetic mechanisms underlying these traits and to provide new and diverse resources for breeders seeking to improve these traits in pea.

The flower color trait used to verify the correct functioning of the PAST analysis model in pea allowed testing of the SNP‐to‐gene assignment function, which worked to assign the large flower color MTA to the correct gene. However, because the causal gene for the flower color trait in this GWAS is a transcription factor, the PAST analysis was not able to assign the gene to a pathway, because transcription factors are not included in the pathways they regulate in pathway annotations. Thus, the flower color data in the present panel could not identify the anthocyanin synthesis pathway that causes pigmentation in pea flowers, and this function of the PAST program remains untested in pea.

Assuming the correct function of the PAST analyses, the metabolic pathway analysis run with the outputs of the BLINK GWAS did identify many pathways significantly associated with seed protein and fat concentrations. For fat, 20 pathways were significantly (*p* < 0.02) associated in the multiyear analysis (Table 5). Seven of these were directly associated with the production or degradation of fatty acids; four with the metabolism or modification of amino acids and proteins; and two pathways act in more upstream steps of the metabolism of both proteins and amino acids. Seven pathways were associated with protein concentration, including one degrading the amino acid L‐leucine, one involved in lipid biosynthesis, and three involved in upstream metabolism of both proteins and lipids. These upstream steps were typically associated with the tricarboxylic acid cycle (TCA, also known as the Krebs cycle or the citric acid cycle), which generates energy via the oxidation of acetyl‐coenzyme A derived from carbohydrates, fatty acids, and proteins. The TCA cycle can also convert intermediate metabolites back to carbohydrates, fatty acids, and proteins, thus increasing one at the expense of the other. The other pathways associated with both fats and proteins were mainly involved in redox or stress responses.

The identification of similar mechanisms in the fat and protein analyses indicates that they may be correlated at the metabolic level. Although the PAST analysis was run on the corrected BLUPs, the results of the fat and protein analyses may still be entwined via the linkage problems identified earlier. While the results have been instructive, considering the large linkage blocks around the flower color gene and some of the other MTAs, the PAST results may suffer from a high false positive rate. Several SNPs were identified by GWAS that were not identified by PAST, either because they were not associated with genes annotated in pathway databases, were in pathways with too few genes to be included in the analysis or did not increase the overall pathway running enrichment score above the significance threshold. The SNPs with the strongest effect on protein (those that accounted for more than 5% of the phenotypic variation) were S1LG6_34341647 and S3LG5_327115929, neither of which were identified by PAST. SNP S7LG7_434876919, with a slightly smaller phenotypic effect, was found via the PAST analysis.

The SNPs with the largest effect on protein that were identified in multiple environments were chosen as candidates for future use in marker‐assisted selection. A total of 47 SNPs can be found in Table S11, along with the allele calls for each of the accessions in the study. While this is more than is generally used for marker‐assisted selection, not all will segregate between the parents of all crosses, so it is important to have multiple possibilities. Because SPC is a quantitative trait, even accessions containing many of the beneficial alleles for these SNPs may not have the highest protein levels; however, many of the 40 accessions found to have the highest protein levels in this study did also have high numbers of beneficial alleles (Table S11). Entries were given the score with the number of beneficial alleles minus the number of detrimental alleles at all SNPs, and this score was correlated to protein level (*R* ^2^ = 0.69). Because none of SNPs were found with all beneficial alleles in a single accession, there is room for improvement via marker‐assisted selection. Donors for these beneficial alleles can be identified in Table S11. The 40 diverse, high‐protein accessions and other donor accessions with specific beneficial SNPs can be used with KASP assays that will be created for the SNPs presented here for marker‐assisted selection of new high‐protein cultivars.

## AUTHOR CONTRIBUTIONS

**Renan Uhdre**: Conceptualization; formal analysis; software; writing—original draft; writing—review and editing. **Clarice J. Coyne**: Conceptualization; methodology; writing—original draft; writing—review and editing. **Britton Bourland**: Methodology; writing—review and editing. **Julia Piaskowski**: Formal analysis; writing—review and editing. **Ping Zheng**: Conceptualization; writing—review and editing. **Girish M. Ganjyal**: Writing—review and editing. **Zhiwu Zhang**: Formal analysis; software; writing—review and editing. **Rebecca J. McGee**: Conceptualization; writing—review and editing. **Dorrie Main**: Conceptualization; writing—review and editing. **Nonoy Bandillo**: Formal analysis; writing—review and editing. **Mario Morales**: Writing—review and editing. **Yu Ma**: Conceptualization; writing—review and editing. **Chengci Chen**: Conceptualization; writing—review and editing. **William Franck**: Methodology; writing—review and editing. **Adam Thrash**: Software; writing—review and editing. **Marilyn L. Warburton**: Conceptualization; formal analysis; software; writing—original draft; writing—review and editing.

## CONFLICT OF INTEREST STATEMENT

The authors declare no conflicts of interest.

## Supporting information


**Supplemental Figure 1**. Marker distribution throughout the pea genome. The number of SNPs distributed along the 7 chromosomes within 1Mb window size in pea genome.

**Supplemental Figure 2**. Admixture plot with *K* = 6. Admixture analysis using all SNPs data to identify the number of *K*.

**Supplemental Figure 3**. Accessions distribution throughout the six groups of the population structure.

**Supplemental Figure 4**. Quantile‐quantile plot (*QQ*‐plot) considering the SNP markers distributions across the pea genome: (a) Protein concentration in 2019, 2020, 2021, and multiyear, and (b) fat concentration in 2019, 2020, 2021, and multiyear.

**Supplemental Figure 5**. Manhattan plots and *QQ*‐plots of *p*‐values for marker‐trait associations analysis with BLINK, FarmCPU, and MLM models for protein and fat concentration in 2019, 2020, 2021, and multiyear.

**Supplemental Figure 6**. Protein variation associated with the six population groups. Boxplot for Protein MY showing the range of protein concentration throughout the population groups.

**Supplemental Figure 7**. Fat variation associated with the six population groups. Boxplot for Fat MY showing the range of fat concentration throughout the population groups.

**Supplemental Table 1**. The 487 accessions MP3 diversity panel lines from USDA pea plant genetic resource collection with yellow cotyledons including cultivars and landraces. Entries used to create the standard curves for protein and fat analyses via NIR are indicated.

**Supplemental Table 2**. Soil analysis and fertility applied. Soil analysis for each environment and their fertility indicate for each year in the field experiment.

**Supplemental Table 3**. Curves for protein and fat developed using 87 accessions from the MP3 diversity panel lines from USDA pea plant genetic resource collection. Mass for each sample, Protein and Fat factors, Nitrogen%, Protein and Fat concentration% results are indicated.

**Supplemental Table 4**. Original BLUPs average over the replications for protein, fat and flower color. Entries with high protein average concentration and their color are indicated.

**Supplemental Table 5**. Pearson correlations calculated between protein, fat, and flower color traits. The correlation values were considered significant (*p* < 0.01) before use flower color as a fixed effect.

**Supplemental Table 6**. Corrected BLUPs average over the replications for protein and fat using flower color as a fixed effect. The outliers in each trait were removed based on the Z test.

**Supplemental Table 7**. Structure analysis of 487 MP3 diversity panel. The origin, type and subpopulation group for each accession are indicated.

**Supplemental Table 8**. MTAs for protein and fat for all year and multiyear from FarmCPU and MLM models. The significance was based on the false discovery rate (FDR) < 0.05 implemented in GAPIT 3.

**Supplemental Table 9**. Linkage distribution and chromosomal statistics information with *r* ^2^ 0.8 across the entire pea genome. The R base function “summary” was used to describe the information of each chromosome through the default LD analysis output obtained by Tassel software.

**Supplemental Table 10**. Significant pathway results for protein and fat concentration for all year and multiyear. The pathway description, gene name, decreasing and increasing model were based on the threshold *p*‐value < 0.05 using PAST 2.0.0 ‐rc.

**Supplemental Table 11**. MTAs suggested for conversion into user‐friendly KASP assays and their score range for each accession based on beneficial alleles. MTAs were selected based on the largest positive and negative effects with *p*‐value < 0.05 in all models. The major, minor, favorable, and unfavorable alleles are indicated. The range from −20 to 15 (max 47) according to the sum of favorable and unfavorable alleles for protein concentration.

## Data Availability

All data generated or analyzed during this study are included in this published article (and its Supporting Information) or on the following websites. The MP3 diversity panel GBS information is available at the National Center for Biotechnology Information (NCBI) website: https://www.ncbi.nlm.nih.gov/sra/PRJNA730349 and the Genomic, Genetic, and Breeding Resources for Pulse Crop Improvement (PulseDB) website: https://www.pulsedb.org/organism/639. The MP3 diversity panel phenotype information is available at the USDA ARS Germplasm Resource Information Network (GRIN) website: https://www.grin‐global.org.
