# Supplementary material for: Association study of crude seed protein and fat concentration in a USDA pea diversity panel
Source: Plant Genome. 2024 Jul 31;18(1):e20485. doi: 10.1002/tpg2.20485 (PMC11726435; doi:10.1002/tpg2.20485)
Supplement: Supplementary file 1 [file TPG2-18-e20485-s002.docx]

**Figure 4.** Quantile-quantile plot (QQ-plot) considering the SNP markers distributions across the pea genome: (a) Protein Concentration Multi-year, 2019, 2020 and 2021, (b) Fat concentration multi-years, 2019, 2020 and 2021. The red line represents the null hypothesis; the dots inside the grey range represent SNP markers with no association (FDR>0.05), and dots outside the grey range represent the candidate markers associated (FDR<0.05) of protein and fat concentration in seeds using the BLINK model.
