# Supplementary figures and images for: Association study of crude seed protein and fat concentration in a USDA pea diversity panel
Source: Plant Genome. 2024 Jul 31;18(1):e20485. doi: 10.1002/tpg2.20485 (PMC11726435; doi:10.1002/tpg2.20485)

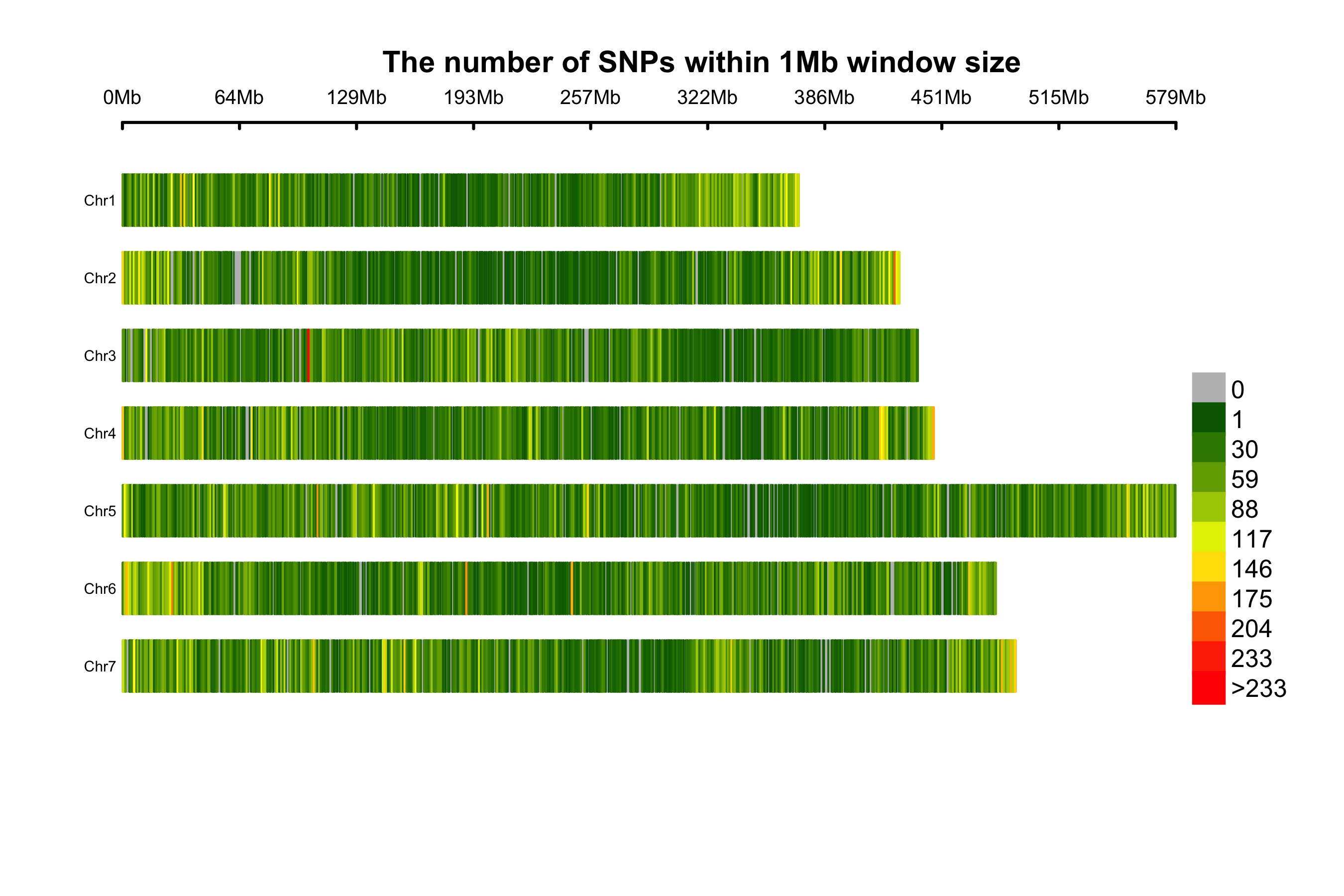

Supplement: Supplementary file 1 — Supplemental Figure 1. Marker distribution throughout the pea genome. The number of SNPs distributed along the 7 chromosomes within 1Mb window size in pea genome. [file TPG2-18-e20485-s018.jpg]

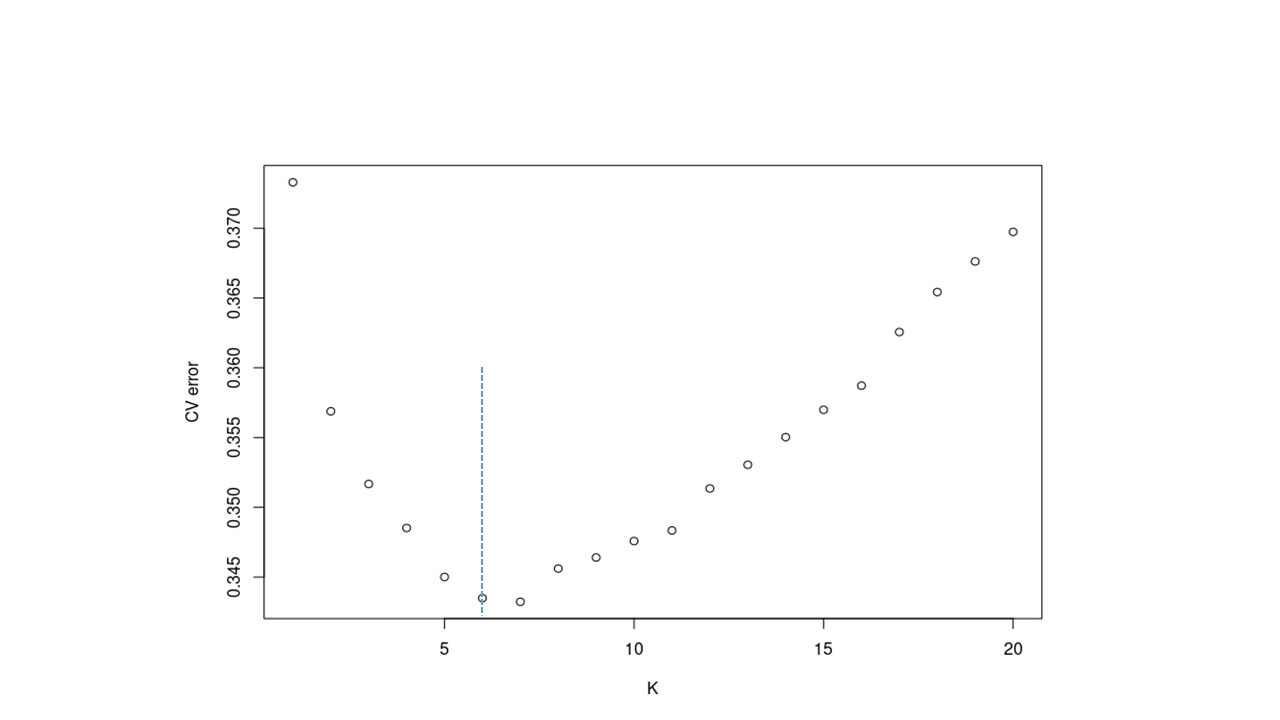

Supplement: Supplementary file 2 — Supplemental Figure 2. Admixture plot with K = 6. Admixture analysis using all SNPs data to identify the number of K. [file TPG2-18-e20485-s016.tif]

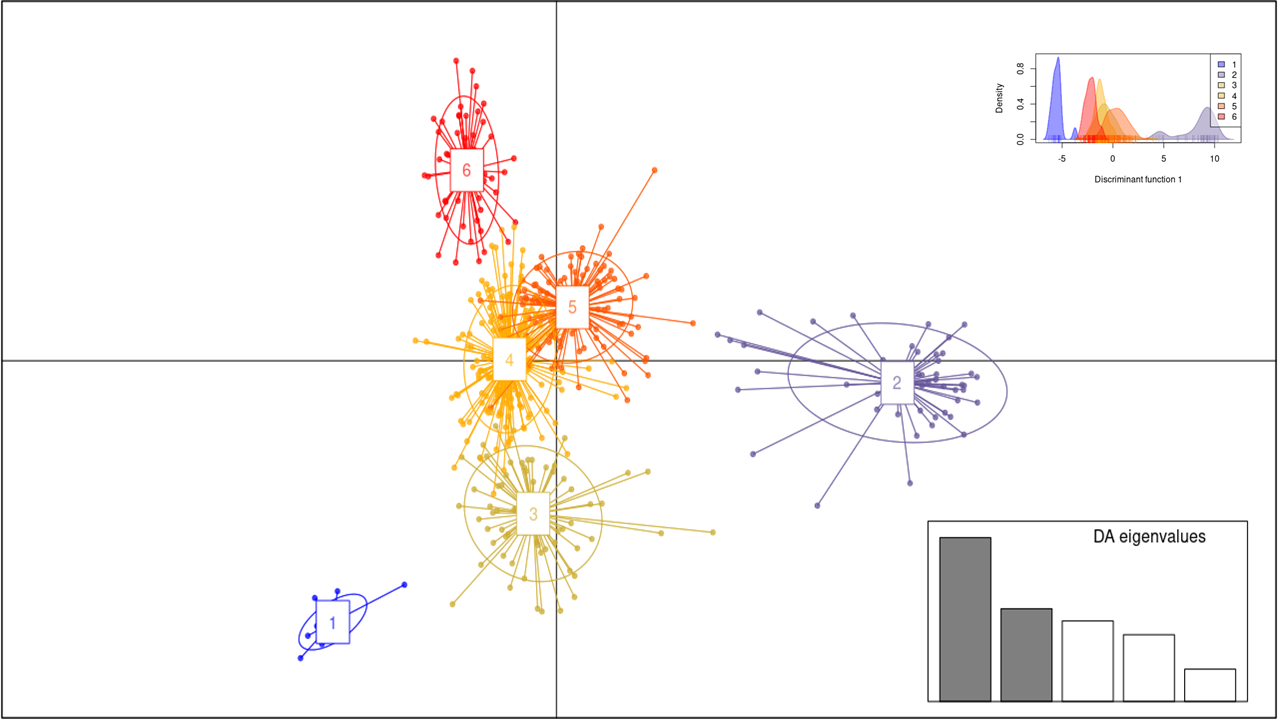

Supplement: Supplementary file 3 — Supplemental Figure 3. Accessions distribution throughout the six groups of the population structure. [file TPG2-18-e20485-s011.tif]

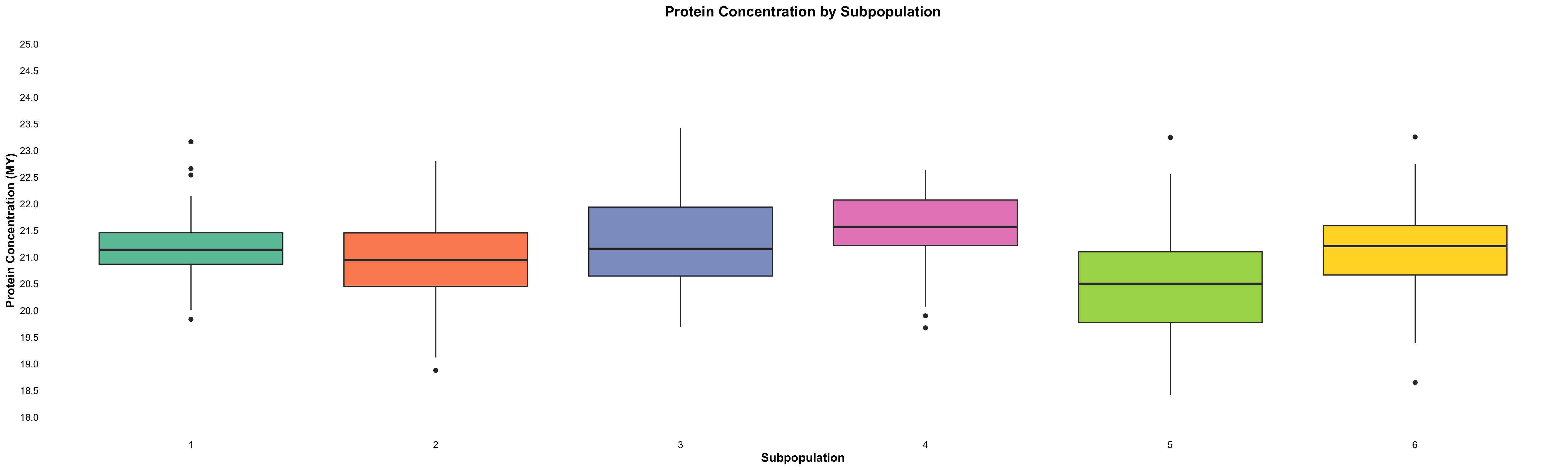

Supplement: Supplementary file 6 — Supplemental Figure 6. Protein variation associated with the six population groups. Boxplot for Protein MY showing the range of protein concentration throughout the population groups. [file TPG2-18-e20485-s005.tiff]

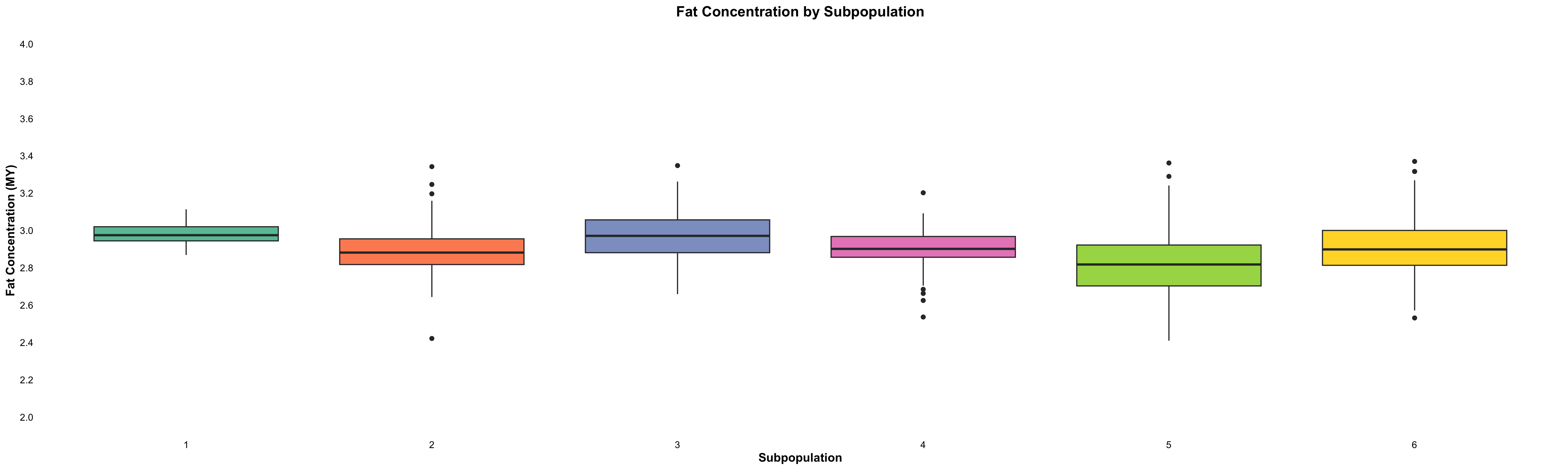

Supplement: Supplementary file 7 — Supplemental Figure 7. Fat variation associated with the six population groups. Boxplot for Fat MY showing the range of fat concentration throughout the population groups. [file TPG2-18-e20485-s007.tiff]
