# Supplementary material for: Association study of crude seed protein and fat concentration in a USDA pea diversity panel
Source: Plant Genome. 2024 Jul 31;18(1):e20485. doi: 10.1002/tpg2.20485 (PMC11726435; doi:10.1002/tpg2.20485)
Supplement: Supplementary file 5 — Supplemental Figure 5. Manhattan plots and QQ‐plots of p‐values for marker‐trait associations analysis with BLINK, FarmCPU, and MLM models for protein and fat concentration in 2019, 2020, 2021, and multiyear. [file TPG2-18-e20485-s010.docx]

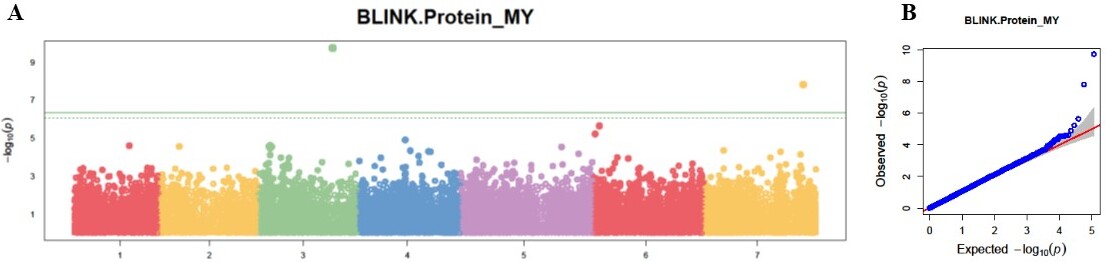


**Figure 5A.** Manhattan plot and QQ-plot of p-values for marker-trait associations analysis with BLINK model for Protein concentration in seeds trait multi-year. In Manhattan plot the vertical axis is the significance of association with the threshold chosen for this study (FDR<0.05), marked by the continuous green line. The horizontal shows the chromosomal location of each single-nucleotide polymorphism. In QQ-plot the red line represents the null hypothesis; the dots inside the grey range represent SNP markers with no association (FDR>0.05), and dots outside the grey range represent the candidate markers associated (FDR<0.05) of protein


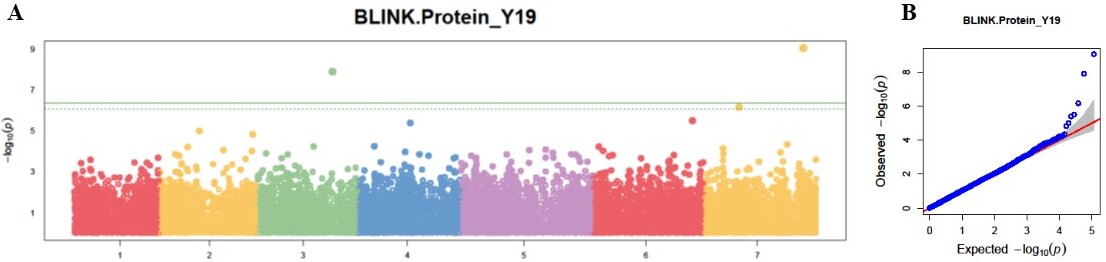


**Figure 5B.** Manhattan plot and QQ-plot of p-values for marker-trait associations analysis with BLINK model for Protein concentration in seeds trait 2019. In Manhattan plot the vertical axis is the significance of association with the threshold chosen for this study (FDR<0.05), marked by the continuous green line. The horizontal shows the chromosomal location of each single-nucleotide polymorphism. In QQ-plot the red line represents the null hypothesis; the dots inside the grey range represent SNP markers with no association (FDR>0.05), and dots outside the grey range represent the candidate markers associated (FDR<0.05) of protein


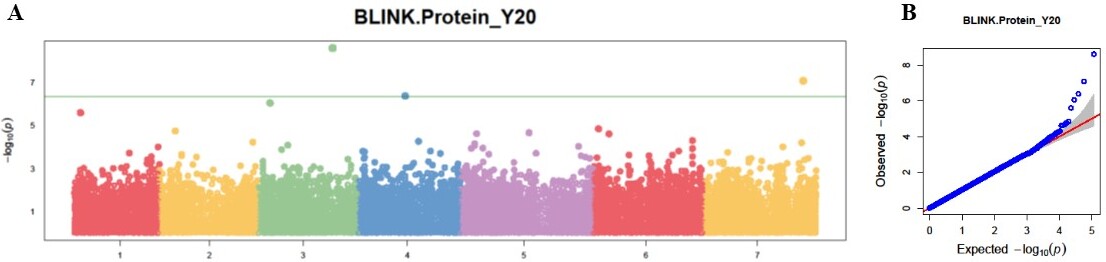


**Figure 5C.** Manhattan plot and QQ-plot of p-values for marker-trait associations analysis with BLINK model for Protein concentration in seeds trait 2020. In Manhattan plot the vertical axis is the significance of association with the threshold chosen for this study (FDR<0.05), marked by the continuous green line. The horizontal shows the chromosomal location of each single-nucleotide polymorphism. In QQ-plot the red line represents the null hypothesis; the dots inside the grey range represent SNP markers with no association (FDR>0.05), and dots outside the grey range represent the candidate markers associated (FDR<0.05) of protein


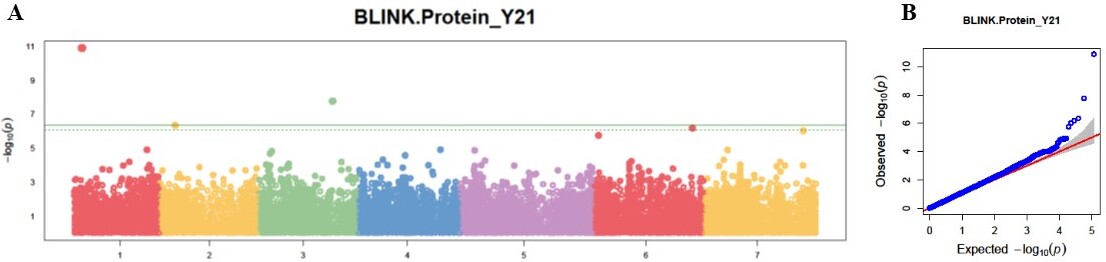


**Figure 5D.** Manhattan plot and QQ-plot of p-values for marker-trait associations analysis with BLINK model for Protein concentration in seeds trait 2020. In Manhattan plot the vertical axis is the significance of association with the threshold chosen for this study (FDR<0.05), marked by the continuous green line. The horizontal shows the chromosomal location of each single-nucleotide polymorphism. In QQ-plot the red line represents the null hypothesis; the dots inside the grey range represent SNP markers with no association (FDR>0.05), and dots outside the grey range represent the candidate markers associated (FDR<0.05) of protein


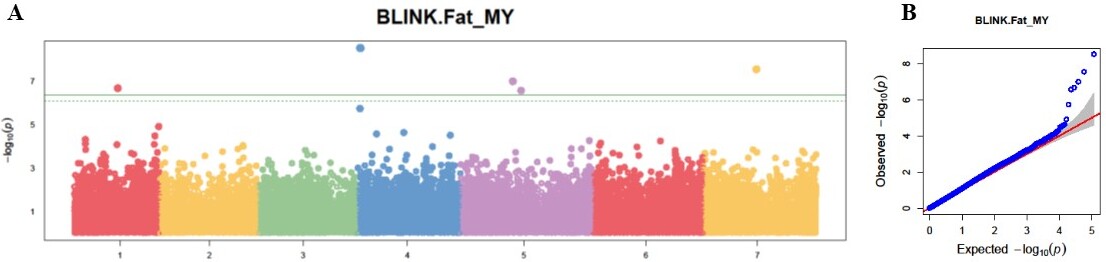


**Figure 5E.** Manhattan plot and QQ-plot of p-values for marker-trait associations analysis with BLINK model for Fat concentration in seeds trait multi-year. In Manhattan plot the vertical axis is the significance of association with the threshold chosen for this study (FDR<0.05), marked by the continuous green line. The horizontal shows the chromosomal location of each single-nucleotide polymorphism. In QQ-plot the red line represents the null hypothesis; the dots inside the grey range represent SNP markers with no association (FDR>0.05), and dots outside the grey range represent the candidate markers associated (FDR<0.05) of fat.


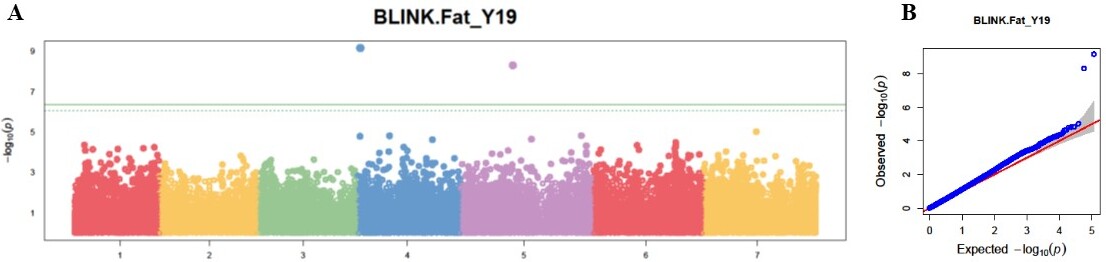


**Figure 5F.** Manhattan plot and QQ-plot of p-values for marker-trait associations analysis with BLINK model for Fat concentration in seeds trait 2019. In Manhattan plot the vertical axis is the significance of association with the threshold chosen for this study (FDR<0.05), marked by the continuous green line. The horizontal shows the chromosomal location of each single-nucleotide polymorphism. In QQ-plot the red line represents the null hypothesis; the dots inside the grey range represent SNP markers with no association (FDR>0.05), and dots outside the grey range represent the candidate markers associated (FDR<0.05) of fat


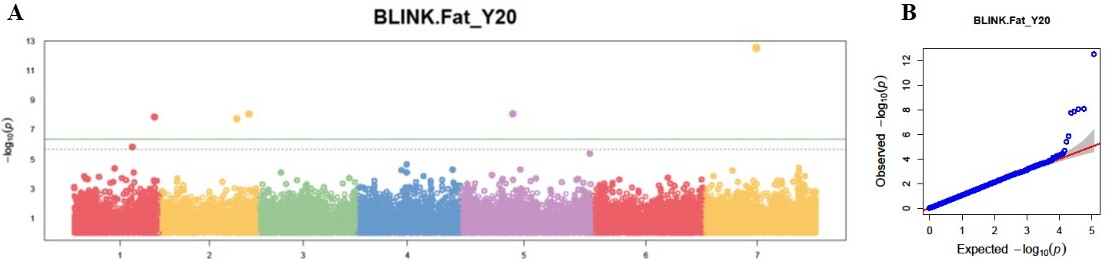


**Figure 5G.** Manhattan plot and QQ-plot of p-values for marker-trait associations analysis with BLINK model for Fat concentration in seeds trait 2020. In Manhattan plot the vertical axis is the significance of association with the threshold chosen for this study (FDR<0.05), marked by the continuous green line. The horizontal shows the chromosomal location of each single-nucleotide polymorphism. In QQ-plot the red line represents the null hypothesis; the dots inside the grey range represent SNP markers with no association (FDR>0.05), and dots outside the grey range represent the candidate markers associated (FDR<0.05) of fat


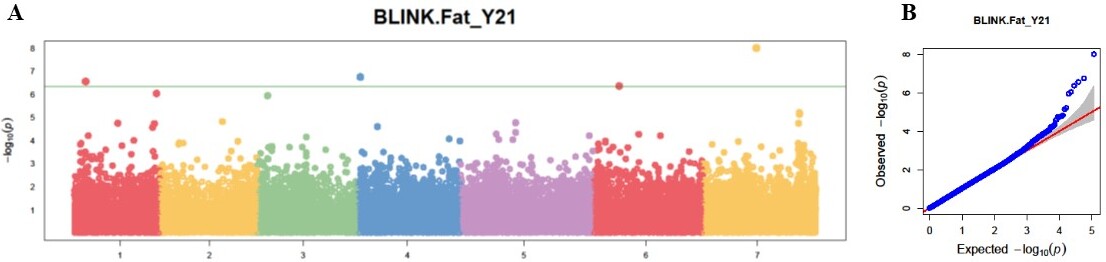


**Figure 5H.** Manhattan plot and QQ-plot of p-values for marker-trait associations analysis with BLINK model for Fat concentration in seeds trait 2021. In Manhattan plot the vertical axis is the significance of association with the threshold chosen for this study (FDR<0.05), marked by the continuous green line. The horizontal shows the chromosomal location of each single-nucleotide polymorphism. In QQ-plot the red line represents the null hypothesis; the dots inside the grey range represent SNP markers with no association (FDR>0.05), and dots outside the grey range represent the candidate markers associated (FDR<0.05) of fat


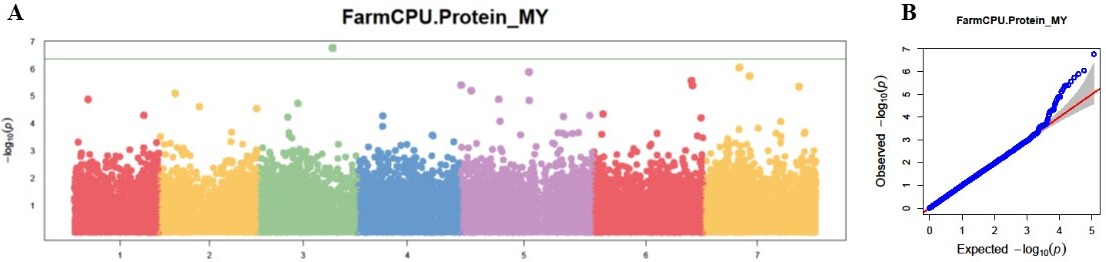


**Figure 5I.** Manhattan plot and QQ-plot of p-values for marker-trait associations analysis with FarmCPU model for Protein concentration in seeds trait multi-year. In Manhattan plot the vertical axis is the significance of association with the threshold chosen for this study (FDR<0.05), marked by the continuous green line. The horizontal shows the chromosomal location of each single-nucleotide polymorphism. In QQ-plot the red line represents the null hypothesis; the dots inside the grey range represent SNP markers with no association (FDR>0.05), and dots outside the grey range represent the candidate markers associated (FDR<0.05) of protein.


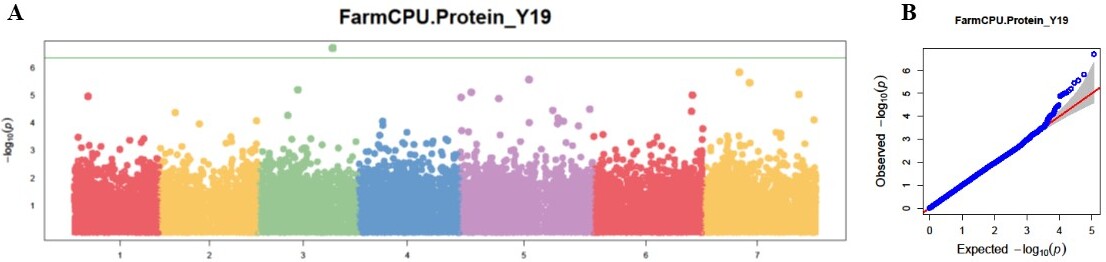


**Figure 5J.** Manhattan plot and QQ-plot of p-values for marker-trait associations analysis with FarmCPU model for Protein concentration in seeds trait 2019. In Manhattan plot the vertical axis is the significance of association with the threshold chosen for this study (FDR<0.05), marked by the continuous green line. The horizontal shows the chromosomal location of each single-nucleotide polymorphism. In QQ-plot the red line represents the null hypothesis; the dots inside the grey range represent SNP markers with no association (FDR>0.05), and dots outside the grey range represent the candidate markers associated (FDR<0.05) of protein.


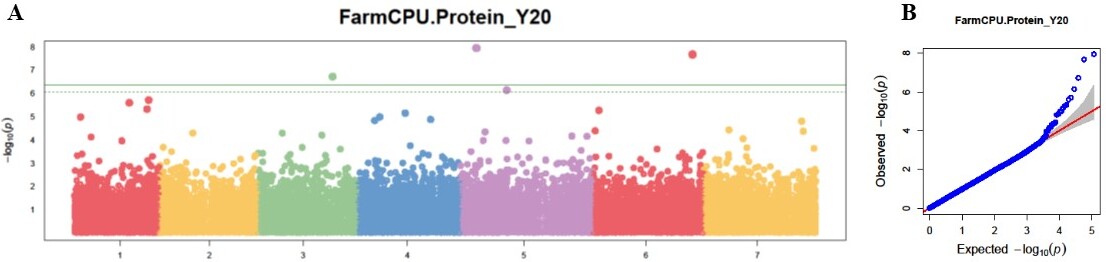


**Figure 5K.** Manhattan plot and QQ-plot of p-values for marker-trait associations analysis with FarmCPU model for Protein concentration in seeds trait 2020. In Manhattan plot the vertical axis is the significance of association with the threshold chosen for this study (FDR<0.05), marked by the continuous green line. The horizontal shows the chromosomal location of each single-nucleotide polymorphism. In QQ-plot the red line represents the null hypothesis; the dots inside the grey range represent SNP markers with no association (FDR>0.05), and dots outside the grey range represent the candidate markers associated (FDR<0.05) of protein.


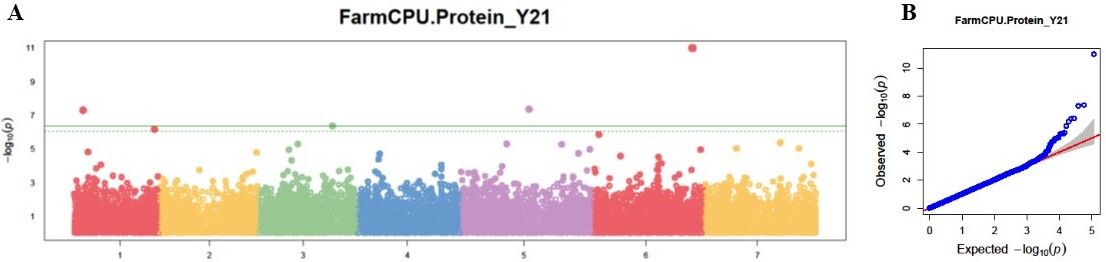


**Figure 5L.** Manhattan plot and QQ-plot of p-values for marker-trait associations analysis with FarmCPU model for Protein concentration in seeds trait 2021. In Manhattan plot the vertical axis is the significance of association with the threshold chosen for this study (FDR<0.05), marked by the continuous green line. The horizontal shows the chromosomal location of each single-nucleotide polymorphism. In QQ-plot the red line represents the null hypothesis; the dots inside the grey range represent SNP markers with no association (FDR>0.05), and dots outside the grey range represent the candidate markers associated (FDR<0.05) of protein.


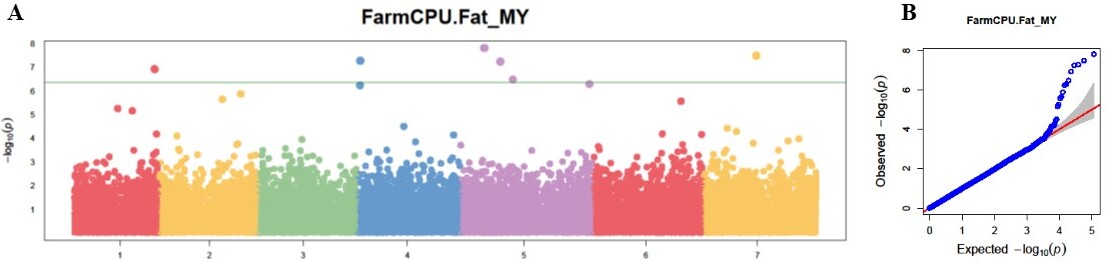


**Figure 5M.** Manhattan plot and QQ-plot of p-values for marker-trait associations analysis with FarmCPU model for Fat concentration in seeds trait multi-year. In Manhattan plot the vertical axis is the significance of association with the threshold chosen for this study (FDR<0.05), marked by the continuous green line. The horizontal shows the chromosomal location of each single-nucleotide polymorphism. In QQ-plot the red line represents the null hypothesis; the dots inside the grey range represent SNP markers with no association (FDR>0.05), and dots outside the grey range represent the candidate markers associated (FDR<0.05) of fat.


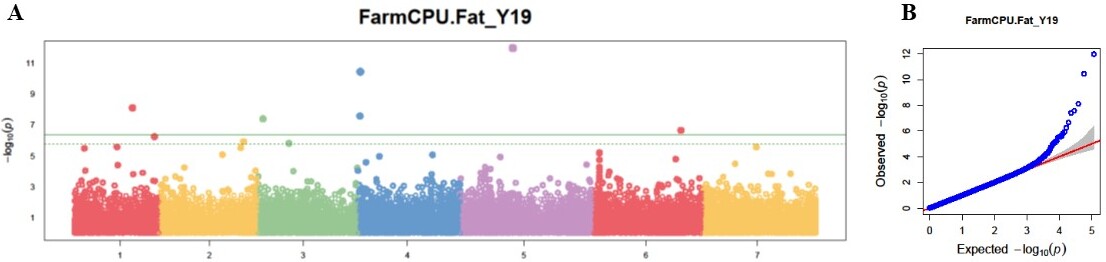


**Figure 5N.** Manhattan plot and QQ-plot of p-values for marker-trait associations analysis with FarmCPU model for Fat concentration in seeds trait 2019. In Manhattan plot the vertical axis is the significance of association with the threshold chosen for this study (FDR<0.05), marked by the continuous green line. The horizontal shows the chromosomal location of each single-nucleotide polymorphism. In QQ-plot the red line represents the null hypothesis; the dots inside the grey range represent SNP markers with no association (FDR>0.05), and dots outside the grey range represent the candidate markers associated (FDR<0.05) of fat.


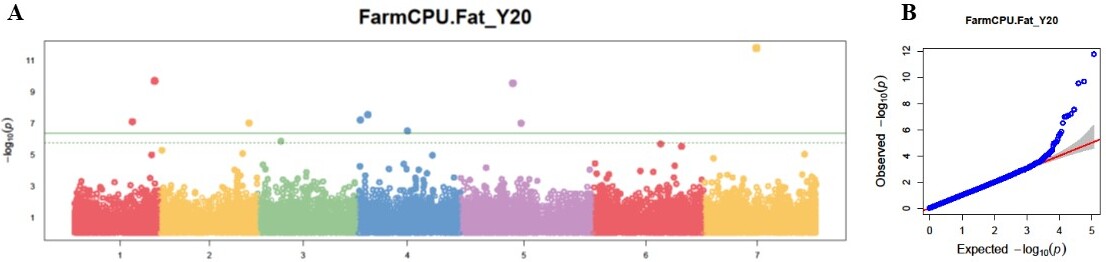


**Figure 5O.** Manhattan plot and QQ-plot of p-values for marker-trait associations analysis with FarmCPU model for Fat concentration in seeds trait 2020. In Manhattan plot the vertical axis is the significance of association with the threshold chosen for this study (FDR<0.05), marked by the continuous green line. The horizontal shows the chromosomal location of each single-nucleotide polymorphism. In QQ-plot the red line represents the null hypothesis; the dots inside the grey range represent SNP markers with no association (FDR>0.05), and dots outside the grey range represent the candidate markers associated (FDR<0.05) of fat.


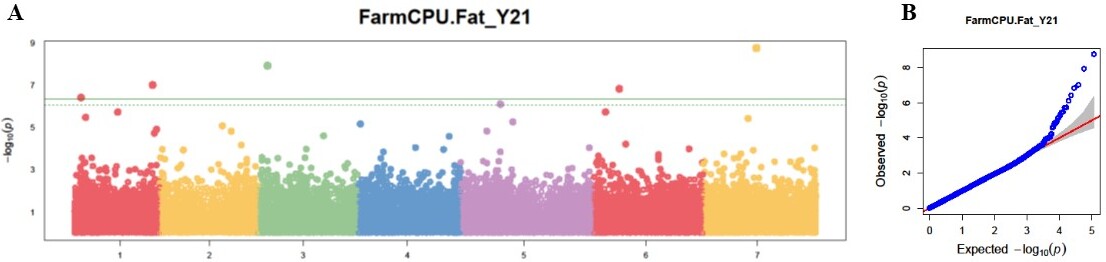


**Figure 5P.** Manhattan plot and QQ-plot of p-values for marker-trait associations analysis with FarmCPU model for Fat concentration in seeds trait 2021. In Manhattan plot the vertical axis is the significance of association with the threshold chosen for this study (FDR<0.05), marked by the continuous green line. The horizontal shows the chromosomal location of each single-nucleotide polymorphism. In QQ-plot the red line represents the null hypothesis; the dots inside the grey range represent SNP markers with no association (FDR>0.05), and dots outside the grey range represent the candidate markers associated (FDR<0.05) of fat.


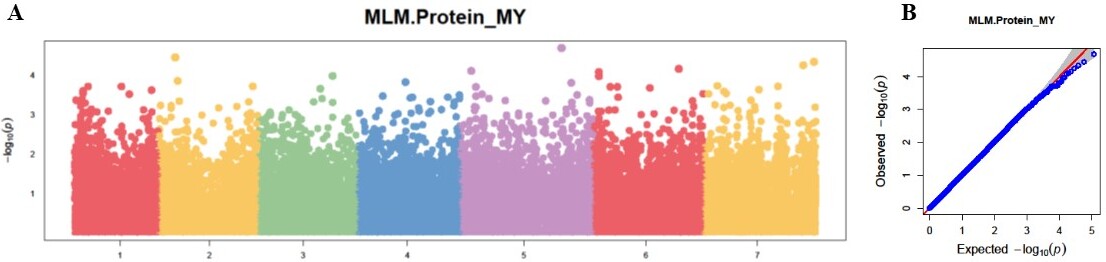


**Figure 5Q.** Manhattan plot and QQ-plot of p-values for marker-trait associations analysis with MLM model for Protein concentration in seeds trait multi-year. In Manhattan plot the vertical axis is the significance of association with the threshold chosen for this study (FDR<0.05), marked by the continuous green line. The horizontal shows the chromosomal location of each single-nucleotide polymorphism. In QQ-plot the red line represents the null hypothesis; the dots inside the grey range represent SNP markers with no association (FDR>0.05), and dots outside the grey range represent the candidate markers associated (FDR<0.05) of protein.


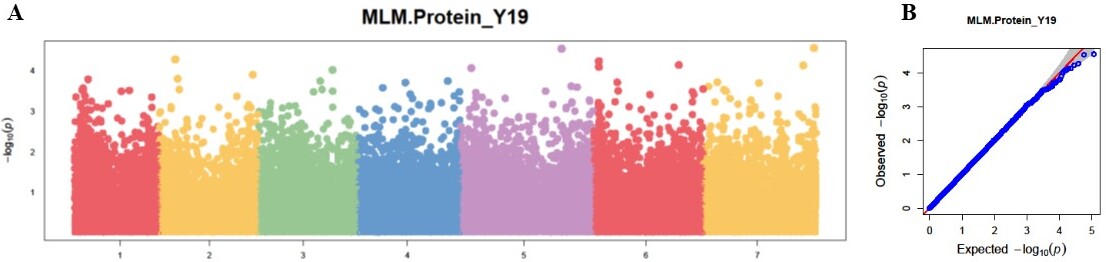


**Figure 5R.** Manhattan plot and QQ-plot of p-values for marker-trait associations analysis with MLM model for Protein concentration in seeds trait 2019. In Manhattan plot the vertical axis is the significance of association with the threshold chosen for this study (FDR<0.05), marked by the continuous green line. The horizontal shows the chromosomal location of each single-nucleotide polymorphism. In QQ-plot the red line represents the null hypothesis; the dots inside the grey range represent SNP markers with no association (FDR>0.05), and dots outside the grey range represent the candidate markers associated (FDR<0.05) of protein.


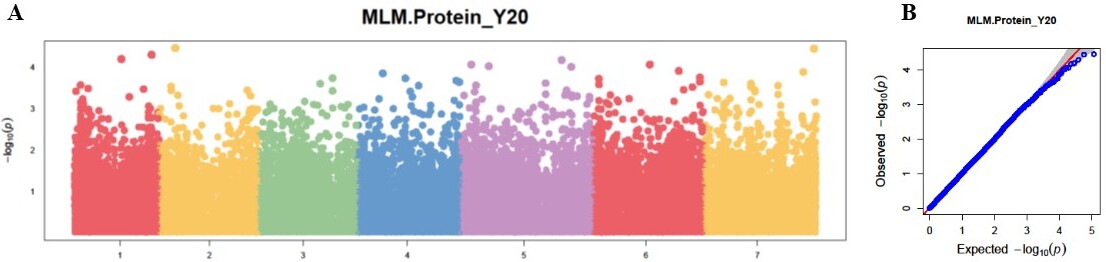


**Figure 5S.** Manhattan plot and QQ-plot of p-values for marker-trait associations analysis with MLM model for Protein concentration in seeds trait 2020. In Manhattan plot the vertical axis is the significance of association with the threshold chosen for this study (FDR<0.05), marked by the continuous green line. The horizontal shows the chromosomal location of each single-nucleotide polymorphism. In QQ-plot the red line represents the null hypothesis; the dots inside the grey range represent SNP markers with no association (FDR>0.05), and dots outside the grey range represent the candidate markers associated (FDR<0.05) of protein.


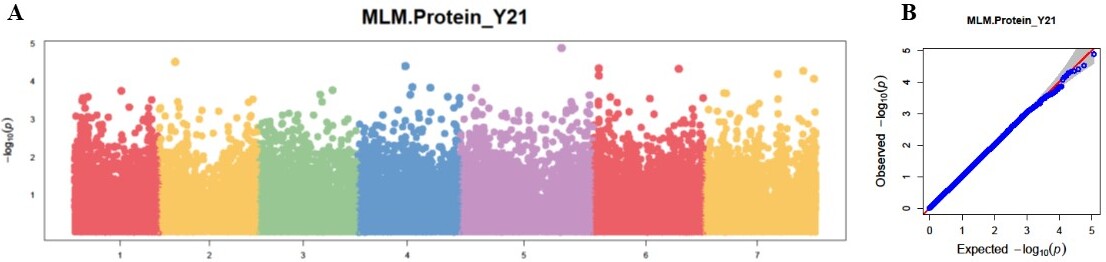


**Figure 5T.** Manhattan plot and QQ-plot of p-values for marker-trait associations analysis with MLM model for Protein concentration in seeds trait 2021. In Manhattan plot the vertical axis is the significance of association with the threshold chosen for this study (FDR<0.05), marked by the continuous green line. The horizontal shows the chromosomal location of each single-nucleotide polymorphism. In QQ-plot the red line represents the null hypothesis; the dots inside the grey range represent SNP markers with no association (FDR>0.05), and dots outside the grey range represent the candidate markers associated (FDR<0.05) of protein.


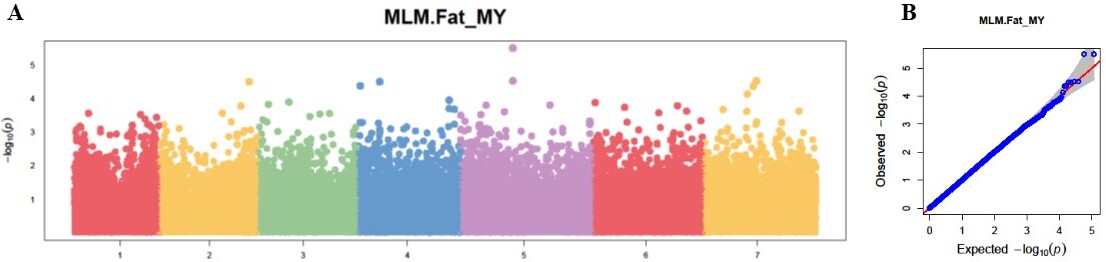


**Figure 5U.** Manhattan plot and QQ-plot of p-values for marker-trait associations analysis with MLM model for Fat concentration in seeds trait multi-year. In Manhattan plot the vertical axis is the significance of association with the threshold chosen for this study (FDR<0.05), marked by the continuous green line. The horizontal shows the chromosomal location of each single-nucleotide polymorphism. In QQ-plot the red line represents the null hypothesis; the dots inside the grey range represent SNP markers with no association (FDR>0.05), and dots outside the grey range represent the candidate markers associated (FDR<0.05) of fat.


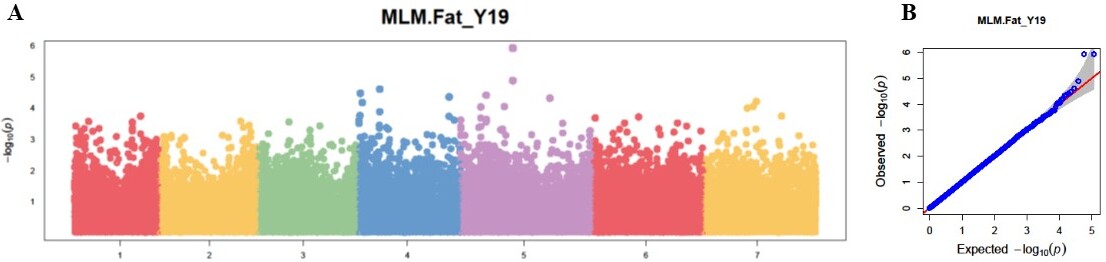


**Figure 5V.** Manhattan plot and QQ-plot of p-values for marker-trait associations analysis with MLM model for Fat concentration in seeds trait 2019. In Manhattan plot the vertical axis is the significance of association with the threshold chosen for this study (FDR<0.05), marked by the continuous green line. The horizontal shows the chromosomal location of each single-nucleotide polymorphism. In QQ-plot the red line represents the null hypothesis; the dots inside the grey range represent SNP markers with no association (FDR>0.05), and dots outside the grey range represent the candidate markers associated (FDR<0.05) of fat.


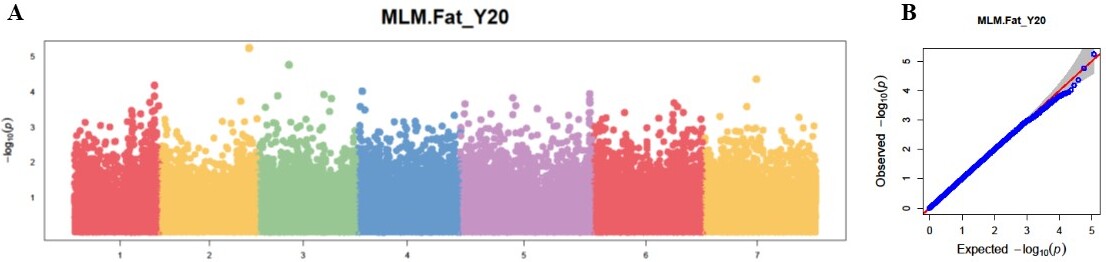


**Figure 5W.** Manhattan plot and QQ-plot of p-values for marker-trait associations analysis with MLM model for Fat concentration in seeds trait 2020. In Manhattan plot the vertical axis is the significance of association with the threshold chosen for this study (FDR<0.05), marked by the continuous green line. The horizontal shows the chromosomal location of each single-nucleotide polymorphism. In QQ-plot the red line represents the null hypothesis; the dots inside the grey range represent SNP markers with no association (FDR>0.05), and dots outside the grey range represent the candidate markers associated (FDR<0.05) of fat.


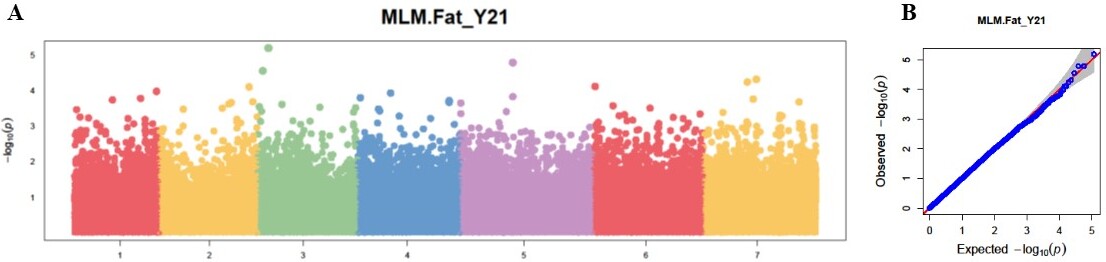


**Figure 5X.** Manhattan plot and QQ-plot of p-values for marker-trait associations analysis with MLM model for Fat concentration in seeds trait 2021. In Manhattan plot the vertical axis is the significance of association with the threshold chosen for this study (FDR<0.05), marked by the continuous green line. The horizontal shows the chromosomal location of each single-nucleotide polymorphism. In QQ-plot the red line represents the null hypothesis; the dots inside the grey range represent SNP markers with no association (FDR>0.05), and dots outside the grey range represent the candidate markers associated (FDR<0.05) of fat.
