# Supplementary material for: Association study of crude seed protein and fat concentration in a USDA pea diversity panel
Source: Plant Genome. 2024 Jul 31;18(1):e20485. doi: 10.1002/tpg2.20485 (PMC11726435; doi:10.1002/tpg2.20485)
Supplement: Supplementary file 12 — Supplemental Table 5. Pearson correlations calculated between protein, fat, and flower color traits. The correlation values were considered significant (p < 0.01) before use flower color as a fixed effect. [file TPG2-18-e20485-s017.docx]

**TABLE S4**. Correlations between the traits measured in the MP3 diversity panel, including protein, fat, and flower color before using the flower color as a fixed effect.

|  | Protein MY | Protein Y19 | Protein Y20 | Protein Y21 | Fat MY | Fat Y19 | Fat Y20 | Fat Y21 | Flower Color |
| --- | --- | --- | --- | --- | --- | --- | --- | --- | --- |
| Protein MY | 1 |  |  |  |  |  |  |  |  |
| Protein Y19 | .86* | 1 |  |  |  |  |  |  |  |
| Protein Y20 | .86* | .56* | 1 |  |  |  |  |  |  |
| Protein Y21 | .88* | .69* | .64* | 1 |  |  |  |  |  |
| Fat MY | -.34* | -.32* | -.19* | -.39* | 1 |  |  |  |  |
| Fat Y19 | -.34* | -.28* | -.21* | -.40* | .99* | 1 |  |  |  |
| Fat Y20 | -.35* | -.33 | -.18 | -.42* | .97* | .94* | 1 |  |  |
| Fat Y21 | -.31* | -.33* | -.17* | -.33* | .98* | .95* | .91* | 1 |  |
| Flower Color | 0.42* | .36* | .30* | .45* | -.89* | -.89* | -.86* | -.86* | 1 |

^*^ correlated at *p≤*0.01
